# CONFINED: distinguishing biological from technical sources of variation by leveraging multiple methylation datasets

**DOI:** 10.1186/s13059-019-1743-y

**Published:** 2019-07-12

**Authors:** Mike Thompson, Zeyuan Johnson Chen, Elior Rahmani, Eran Halperin

**Affiliations:** 1 Department of Computer Science, University of California Los Angeles, Los Angeles, CA USA; 2 Department of Human Genetics, University of California Los Angeles, Los Angeles, CA USA; 3 Department of Anesthesiology and Perioperative Medicine, University of California Los Angeles, Los Angeles, CA USA; 4 Department of Biomathematics, University of California Los Angeles, Los Angeles, CA USA

## Abstract

Methylation datasets are affected by innumerable sources of variability, both biological (cell-type composition, genetics) and technical (batch effects). Here, we propose a reference-free method based on sparse canonical correlation analysis to separate the biological from technical sources of variability. We show through simulations and real data that our method, CONFINED, is not only more accurate than the state-of-the-art reference-free methods for capturing known, replicable biological variability, but it is also considerably more robust to dataset-specific technical variability than previous approaches. CONFINED is available as an R package as detailed at https://github.com/cozygene/CONFINED.

**Electronic supplementary material:**

The online version of this article (10.1186/s13059-019-1743-y) contains supplementary material, which is available to authorized users.

## Introduction

While technological advances have provided a surplus of methylation datasets, analyses of these datasets are often complicated by innumerable possible sources of variability [1, 2]. In particular, epigenome-wide association studies (EWAS) and studies that aim to implicate observed methylation signal to phenotypic variance are particularly at risk for false associations due to unknown drivers of the observed signal that globally affect the epigenome [3–5]. For example, age is correlated with a large number of methylation sites and phenotypes [6–8], and thus if not corrected for, association between a specific methylation site and a phenotype may be primarily driven by a confounder such as age. In order to mitigate spurious associations in such association studies, it is crucial to elucidate and account for the sources of variation that globally affect the methylation patterns in the genome.

Sources of global methylation effects can be either technical or biological and may also be measured or unmeasured. In the case of technical sources, most typical are batch effects, or variation resulting from different technicians or conditions during the data-preparing steps [9]. These sources should undoubtedly be identified and accounted for in analyses, for example, by balancing cases, controls, and samples from different datasets, including measured potential confounders as covariates, regressing out the sources of confounding signals if they are measured, or otherwise estimating these potential sources of technical effects and accounting for their estimates [10].

The case of biological sources is more complex; biological sources of variation such as age, sex, cell-type composition, genetics, ethnicity, co-morbidities, or responses to environmental factors like medication intake or smoking status indeed affect the global methylation patterns in the genome, and they are also often correlated to the phenotype of interest[6, 11–15]. However, due to logistical limitations, often only a few of these sources of biological variation are measured in a given study; moreover, it is often the case that some of the sources of variation that are correlated with the phenotype are unknown and hence unmeasured.

Unlike technical effects, there is much debate over the best practice of using these biological sources of variation in a model (e.g., [3, 13, 16, 17]) since one can argue that identifying these sources is an important ingredient in understanding the disease mechanism. Moreover, identifying these biological sources of variation may be useful in prediction algorithms related to the studied phenotype. In other words, it is context-specific whether one should include biological sources of variation in their model—considering the additional sources as confounders—or simply derive a model considering only the observed signal and accounting for the technical effects[18].

To capture signal corresponding to specific biological sources of variation, reference-based methods have been proposed. In the case of methylation, one commonly researched source of biological variability is cell-type composition. Houseman et al. developed an approach to estimate the true cell-type proportions in methylation datasets using “methylation signatures” (estimates of cell-type-specific methylation levels across a population) [19]. Reference-based methods and methods that leverage prior statistics, however, are limited to known sources of variability for which such reference data exists. In many cases, either the sources of variability are unknown, or there is no reference data that can be utilized for these methods (e.g., factors such as diet and exposure to air pollution [20–22] and tissues such as solid tumors or adipose [23]). In such cases, reference-based methods cannot be used.

**Fig. 1:**
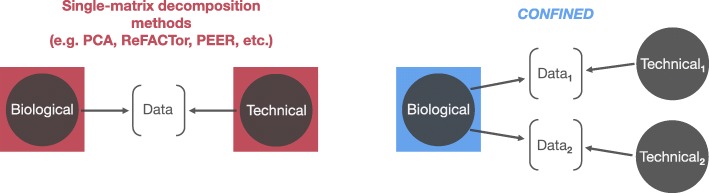
*CONFINED* compared to previous factorization approaches. Previous reference-free methods based on single-matrix decompositions (e.g., principal component analysis, non-negative matrix factorization) capture the dominant sources of variability which may be composed of both biological and technical effects (left). Here, we propose a method to capture solely biological variability (right)

In an attempt to overcome the above limitations, many reference-free methods [23–29] have been proposed. Though these methods can correct for cell-type composition in EWAS [27, 30] and may also capture other sources of variability, they are limited by the fact that it is impossible to know whether their components reflect biological or technical signal (Fig. 1). While technical signal is not of interest and should be accounted for in the analysis, the biological signal can provide useful insights about underlying biological phenomena, for instance by being used to model the interaction with the methylation signal.

In this paper, we propose a reference-free method that disentangles the technical sources of variation from the biological sources of variation. Our method is based on the observation that the same biological sources of variation typically affect different studies that are performed under the same conditions (e.g., on the same tissue type), while technical variability is study-specific. Thus, unlike previous unsupervised methods that utilize single-matrix decomposition techniques to account for covariates in methylation data, we propose the use of canonical correlation analysis (CCA), which captures shared signal across multiple datasets. In brief, CCA finds shared structure between two datasets by finding maximally correlated linear transformations of the datasets and is used across many fields including cognitive science[31], psychology[32], and imaging[33]. CCA has been used in the context of genomics to capture genome-wide similarities between different genomic measurements (e.g., gene expression and genetics [34, 35], gene expression and copy number alterations [36, 37]) for the same set of individuals. As opposed to this traditional use of CCA, our method, named *CONFINED* (CCA ON Features for INter-dataset Effect Detection), searches for genome-wide similarities between one methylation profile across two sets of individuals. By instead searching across a single genomic profile, we capture shared structure inherent to the underlying biology of the datasets.

The key discrepancy between *CONFINED* and previous reference-free methods is that *CONFINED* will only capture shared sources of variability. There are two notable examples in which a method like *CONFINED* can be leveraged over unsupervised methods that capture dataset-specific variability. First, when capturing unmeasurable and unknown sources of variability, *CONFINED* will distinguish between the technical and biological components of such sources, as technical variability tends to be dataset-specific. Second, if the goal of a study was to elucidate the effects of a dataset-specific effect such as a treatment effect, and one wished to capture and control for covariates, single-matrix methods would fail and adjust away the effect of interest. In short, one could include the components generated by CONFINED to model their effects or interaction with the methylation, in for example, an EWAS, or instead remove the signal captured by the components prior to studying dataset-specific variability such as a treatment effect on a subset of individuals.

We evaluated the performance of *CONFINED* through both simulated and real data. Our evaluations demonstrate that *CONFINED* captures signal from only biologically replicable sources of variability. We show, as examples, improvement over previous methods by comparing their performance in capturing methylation signal due to known, measurable sources of variability such as cell-type composition, age, and sex in several whole-blood datasets. We also demonstrate that by inducing sparsity, *CONFINED* prioritizes features that recapitulate biological functionality inherent to both datasets. For example, when pairing two whole-blood datasets together, the sites best ranked by *CONFINED* were significantly enriched for immune cell function.

## Results

### A brief summary of *CONFINED*

We developed *CONFINED* to capture biological sources of variability in methylation datasets. As input, *CONFINED* takes two matrices with the same number of rows (methylation sites) but not necessarily the same number of columns (individuals), *k* the number of components to produce, and *t* the number of CpG sites to use, or in other words, a sparsity parameter. As output, *CONFINED* produces *k* components that can be used to model biological sources of variability for each input dataset.

Notably, *CONFINED* is based on CCA which considers two datasets simultaneously. Intuitively, CCA performs a decomposition of two matrices simultaneously and hence finds linear combinations of features that define biological variation present in both datasets. Conversely, previous methods that decompose one matrix at a time essentially look for linear or non-linear (kernel-based) combinations of features that preserve dominant structure in a single dataset, and this structure may be a combination of both biological and technical signal. Thus, leveraging the shared structure of two datasets through CCA is crucial. Nonetheless, there are two substantial differences between *CONFINED* and traditional uses of CCA in genomic studies. First, *CONFINED* looks for shared structure of one methylation profile across two sets of individuals rather than looking for shared structure in one set of individuals across two sets of genomic measurements. Second, *CONFINED* performs a feature selection procedure that is critical to detect the shared sources of variability across the different datasets.

### *CONFINED* finds biological sources of variability with high accuracy: analysis across datasets of the same tissue type

We first evaluated *CONFINED* using a pair of whole-blood methylation datasets from Hannum et al. [38] and Liu et al. [39]. Along with their methylation data were measured sources of biological variation including patients’ disease status, age, and sex. In addition to evaluating *CONFINED*’s ability to capture the measured biological factors, we also evaluated its performance on an unmeasured source of variation, cell-type composition. While in this section, we focused on using two datasets corresponding to the same tissue type, we note that the studied phenotypes in the datasets were different (e.g., Hannum et al. studied aging whereas Liu et al. studied Rheumatoid arthritis). As *CONFINED* looks for only *shared* biological sources of variation, we excluded from our evaluations sources of variation that may only appear in one of the datasets, e.g., patient status. As we show below, using *CONFINED* we were able to produce components that correlated with both the measured and unmeasured sources of biological signal across both datasets. We also evaluated the ability of *CONFINED* to generate components correlated with several measured shared sources of variability on a pair of adipose datasets as well as a pair of brain tissue datasets (Additional file 1: Figures S1 and S2).

**Fig. 2:**
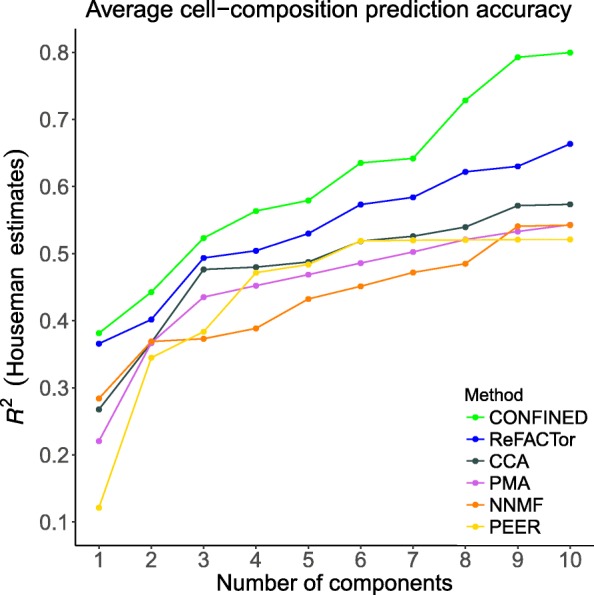
A comparison of *CONFINED* and previous reference-free methods in capturing leukocyte composition. We used each methods’ components to capture cell-type proportions as estimated by the reference-based method of Houseman et al. [19] across CD4 T cells, CD8 T cells, monocytes, B cells, natural killer cells, and granulocytes in whole-blood data from an aging study (here; Additional file 1: Figure S3 Hannum et al. [38]) as well as in whole-blood from a study of Rheumatoid arthritis (Liu et al. [39] Additional file 1: Figure S4)

First, we evaluated *CONFINED* against other reference-free methods when capturing unmeasured biological sources of variability in two whole-blood datasets. Here, we used *CONFINED* to capture cell-type composition, which was unmeasured in both studies. We treated cell-type proportion estimates from the reference-based algorithm of Houseman et al. [23] as the ground-truth. Houseman et al. proposed a reference-based method for estimating proportions of immune cells in whole-blood methylation data by leveraging differentially methylated regions of DNA to form methylation signatures for individual cell-types. They then use these signatures to obtain cell proportion estimates for several immune cells (CD4 T cells, CD8 T cells, B cells, natural killer cells, monocytes and granulocytes). In our experiments, we fit a linear model of each Houseman-estimated cell-type proportion using several components from each of the methods. *CONFINED* outperformed all of the previous methods we tested, with pronounced differences in its estimation of the composition of monocytes and natural killer cells (Fig. 2, Additional file 1: Figures S3, S4, S5 and S6. To clarify if the gain in performance was a result of *CONFINED* using more individuals or a more informative feature selection, we considered the situation in which two datasets are concatenated and supplied to a single-matrix-decomposition method as a single dataset, as well as the situation in which a single-matrix decomposition method leverages the features selected by *CONFINED*. In both procedures, however, the components of the single-matrix method were less correlated to cell-type composition than the components of *CONFINED* (Additional file 1: Figures S7 and S8).

**Fig. 3:**
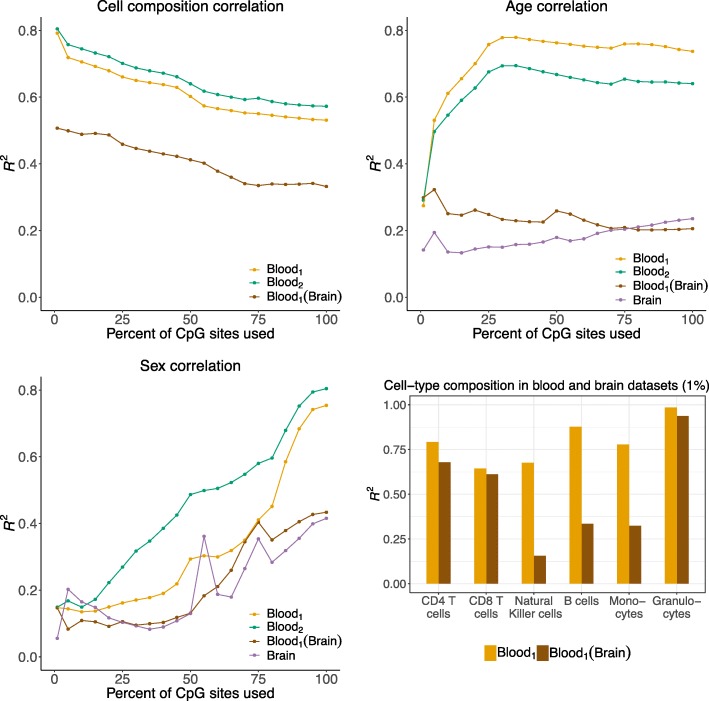
Biological drivers of variability captured by *CONFINED* across a range of sparsity. We paired a whole-blood dataset [39] with another whole-blood dataset [38] and with a brain dataset [43] to capture sources of variability in each dataset. We fit a linear model for each source of variability using 10 *CONFINED* components to obtain an *R*^2^ value. We varied the percentage of CpG sites used from 1% (nearly entirely sparse) to 100% (no sparsity)

We next considered the ability of *CONFINED* when searching for known, measured sources of variability. For the same pair of blood datasets *CONFINED*’s components captured age and sex with accuracy $R^{2}_{\text {age}} >.74$ and $R^{2}_{\text {sex}} >.70$ respectively (Fig. 3). In the case of other methods, PMA [36] had the highest performance among previous methods, but was greatly outperformed by *CONFINED* (Additional file 1: Figure S9). Notably, using relatively less sparsity to capture age and sex achieved greater accuracy, however this trend was not necessarily observed when using lower sparsity for capturing cell-type composition.

To better understand the implications of *CONFINED*’s sparsity parameter, we evaluated the biological significance of the features selected by *CONFINED* using the R package missMethyl| [40]. For a given set of methylation sites, missMethyl tests for enrichment in gene ontology (GO) pathways by first mapping the sites to genes (weighing the genes based on the number of sites that map to them), then performing a test built off of Wallenius’ noncentral hypergeometric distribution. In order to avoid potential biases resulting from the parametric assumptions in the model of missMethyl, we performed permutation testing using its reported p-values. Our test yielded significant enrichment for various ontologies across multiple pairs of datasets (Table 1; Additional file 1: Table S1, Table S2, Table S3). When we paired two whole-blood datasets, the highest ranked features by *CONFINED* were enriched for pathways generally involved with the immune response, leukocyte activation, and defense response. Notably, most of the significantly enriched pathways were related to the immune system or signaling (Table 1). When looking at the enrichment for adipose and brain tissues, we saw pathways concerning vascularization and sheathing respectively. These results underscore the importance of *CONFINED*’s sparsity and provide support for *CONFINED*’s ability to capture biologically meaningful signal, such as tissue-specific cell-type functions.

**Table 1:** Gene Ontology enrichment of sites ranked by *CONFINED*

Ontology term	p-value (permutation)	p-value (missMethyl)
Immune system process	.001	6.9e−18
Immune response	.001	1.0e−15
Regulation of immune response	.026	3.0e−11
Defense response	.038	7.18e−11
Regulation of immune system response	.039	7.18e−11
Response to external biotic stimulus	.059	2.58e−10
Response to other organism	.059	2.58e−10
Leukocyte activation	.069	4.68e−10
Regulation of immune effector process	.090	1.86e−09
Response to biotic stimulus	.095	2.46e−09
Positive regulation of immune system process	.100	2.89e−09
Response to bacterium	.103	3.65e−09
Cell activation	.104	3.77e−09
Immune effector process	.104	3.77e−09
Response to stress	.136	1.77e−08
Lymphocyte activation	.139	1.25e−08
Positive regulation of immune response	.143	1.49e−08
Regulation of leukocyte activation	.145	1.59e−08
Regulation of cell activation	.185	2.91e−08
Protein binding	.190	3.10e−08

We tested enrichment of the highest-ranked sites by *CONFINED* in a blood-blood pair of datasets. Here, we set the sparsity parameter based on a rule learned through cross-validation ; however, we observed qualitatively similar results across a range of sparsity parameters, with increasing significance when we included a relatively larger number of CpG sites (Additional file 1: Figure S11)

### *CONFINED* distinguishes between dataset-specific and shared signal: Real data analysis with simulated dataset-specific effects

In the context of capturing biological signal, one of the main limitations of single-matrix decomposition methods (e.g., PCA, ReFACTor [24], PEER [41], non-negative matrix factorization (NNMF) [42]) is that each of their components may consist of a mixture of signal reflective of technical noise specific to a dataset, such as batch effects, and the biological signal. For instance, PCA and methods based on PCA, such as ReFACTor [24] and penalized matrix decomposition (PMA) [36], consider directions in the data that explain the most variability, but this variability is not limited to strictly global biological or replicable effects in the individual datasets. This issue may also be present in PEER [41], which includes a probabilistic version of factor analysis, as the latent factors driving the data may also include some effect from technical variability. Similarly, in NNMF [42], a data matrix is decomposed as a linear combination of different components, and some of the signal of the data matrix may be deconstructed by a component that captures technical variation. Intuitively, *CONFINED* should be robust to dataset-specific technical effects as it only looks for shared structure across datasets.

**Fig. 4:**
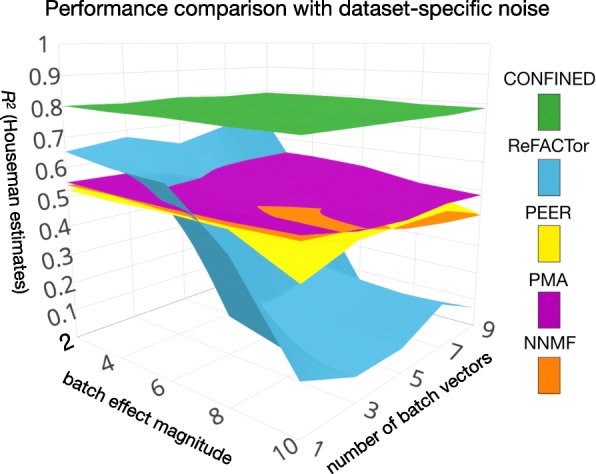
Capturing cell-composition in the presence of simulated technical noise. We added simulated batch effects to the whole-blood datasets of Liu et al. [39] and Hannum et al. [38] and compared the ability of *CONFINED*, ReFACTor[24], PEER[41], PMA[36], and NNMF to capture cell-type composition in whole-blood. Here, we show the results of the Hannum et al. dataset; however, the results of each method were quantitatively similar across both datasets (Additional file 1: Figure S12)

To illustrate that *CONFINED* captures only replicable biological signal, we simulated batch effects for two whole-blood methylation datasets from Hannum et al. [38] and Liu et al. [39] and compared our method to several earlier methods based on single-matrix decomposition. In this setting, we generated dataset-specific noise with low-rank structure and added it to each of the datasets prior to running any feature selection or method. Naturally, simulated batch effects induce technical variation in the datasets, and thus may interfere with methods’ abilities to capture biological variation. We used the datasets with added noise to capture cell-proportion estimates of the original datasets as reported by the method proposed by Houseman et al. [19] (Fig. 4).

We evaluated the performance of each method while varying the strength of simulated, dataset-specific technical effects (Methods, Additional file 1: Figure S12). The components of *CONFINED* best captured the biological signal and were the only components that were robust to technical variation across all levels of noise (Fig. 4). In addition to the biological signal, the components of the previous methods captured signal pertaining to the simulated batch effects (Additional file 1: Figure S13).

We also considered the scenario in which a preprocessing step is taken prior to running each method in order to remove technical variation or noise. Here, we used Remove Unwanted Variation (RUV) [2, 9] to generate components which we regressed out from the datasets with added noise prior to running any of the previous methods (Additional file 1: Figure S14). Using RUV as a preprocessing step helped improve the single-matrix methods in the presence of simulated technical noise, however the components generated by *CONFINED* in the presence of the technical noise (and without any such preprocessing) were still more correlated with cell-type composition than those produced by the single-matrix methods (Additional file 1: Figure S14).

**Fig. 5:**
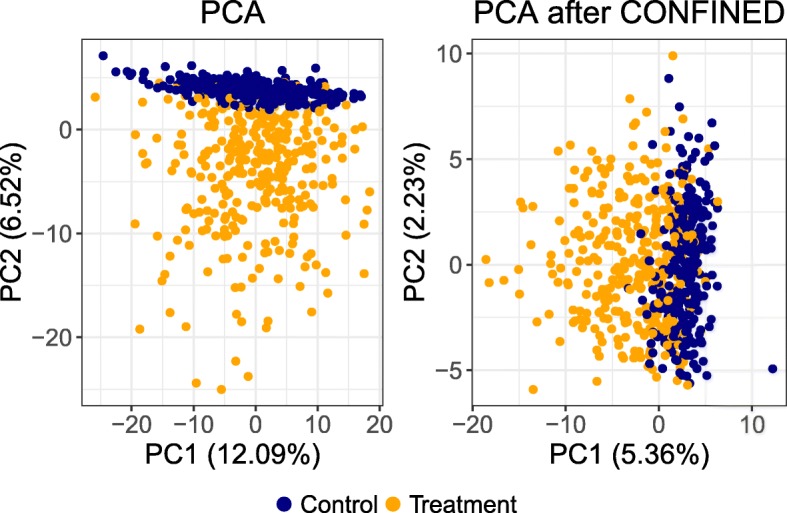
Highlighting treatment effect. We removed from a dataset with simulated treatment effect the components generated by *CONFINED*. Notably, this simulated treatment effect was not shared across datasets. On the left, PCA performed on the dataset prior to removing the *CONFINED* components, and on the right the PCA of the dataset after regressing out the *CONFINED* components

In the case where one wishes to elucidate the effects of a treatment that has been administered to a set of individuals in one dataset, *CONFINED* may also be of use. In a second simulation experiment, we simulated a rank-one treatment effect following a similar strategy used in the batch effects simulations (“Methods” section), only that we used the absolute value of the batch effect scores (i.e., we assumed that the treatment effect had the same directionality across samples). We then added this positive treatment effect to a subset of individuals in one of the whole-blood datasets prior to any analysis. We paired the dataset with added treatment effects with one of the raw datasets and obtained the *CONFINED* components for each dataset. Afterward, we regressed out the top 10 *CONFINED* components from the treatment dataset. Comparing the PCA plots of the treatment dataset before and after preprocessing (i.e., removing the shared signal) shows how *CONFINED* can be leveraged to highlight a dataset-specific treatment effect (Fig. 5). In the scenario where the treatment effect was a dominant source of variability, using *CONFINED* as a preprocessing step did not obstruct the ability to distinguish between those who received treatment and those who did not (the correlation between the treatment group and the first two PCs changed from.429 to.414).

### *CONFINED* finds the shared biology across datasets: analysis of datasets of different tissue types

We also used *CONFINED*’s components to capture measured sources of biological variation across tissue-types (Fig. 3). In one experiment, we paired a whole-blood dataset [39] with a dataset from Lunnon et al. [43] composed from brain tissue. Notably, the accuracy of *CONFINED* to capture each source of signal varied depending on the pairing of the tissue-type (i.e., blood-blood vs. blood-brain) and the sparsity parameter used.

When pairing the blood dataset with the brain dataset, *CONFINED*’s components were correlated with some of the whole-blood dataset’s measured biological factors with slightly less strength than when pairing it with a dataset of the same tissue type ($R^{2}_{\text {age}} >.27, R^{2}_{\text {sex}} >.39$) (Fig. 3), possibly suggesting a different architecture for genome-wide variation across the different tissue types. Nonetheless, the cell-type composition accuracy for the blood dataset when paired with the brain dataset was still relatively high (average $R^{2}_{\text {cell}} =.54)$. This is likely due to the fact that several types of immune cells are known to populate or have immune-related functions in the brain (e.g., resident T cells [44, 45], glia [46] and neutrophils (granulocytes)[47]). Therefore, the immune function of cells in the brain and immune cells in the blood may follow similar pathways that could be reflected in the epigenome. The biological sources of variability in the brain dataset were captured with overall less accuracy than the whole-blood biological sources of variability ($R^{2}_{\text {age}} >.21, R^{2}_{\text {sex}} >.33$).

When pairing the blood and brain datasets, we observed enrichment results somewhat similar to when using the blood-blood pair, but with less significance. The most enriched pathways in the blood-brain pair included several immune system or hematopoietic processes, but the less enriched pathways were primarily different than when pairing the two blood datasets. The pathways in the blood-brain pair were generally not significantly enriched using permutation testing, unless we used a relatively lower level of sparsity.

**Fig. 6:**
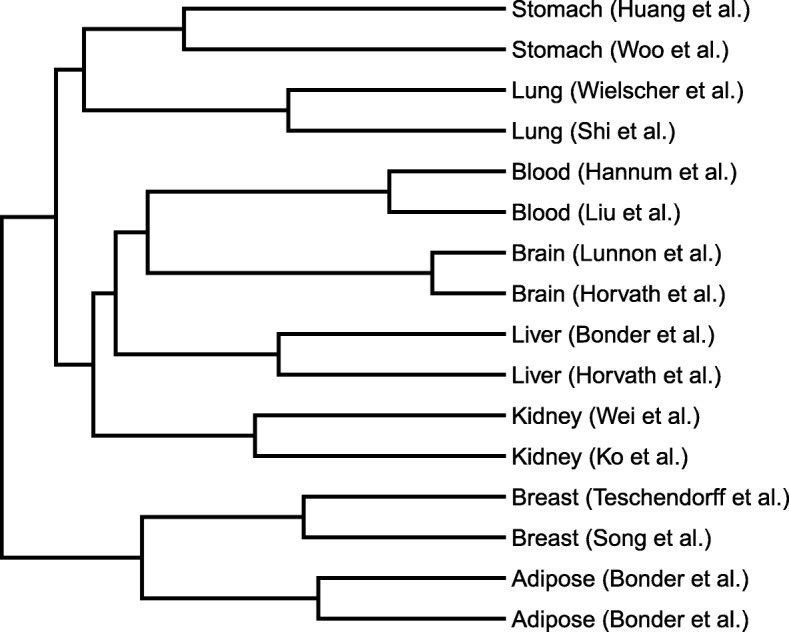
Capturing shared biology across datasets. To validate that *CONFINED* finds biology shared across datasets, we gathered 2 datasets for 9 tissue types, then considered their CCA-based correlations as a metric of similarity. Here, we perform hierarchical clustering, using as a metric of similarity the mean of the top 10 CCA-based correlations

Considering *CONFINED*’s ability to find the biological signal shared across two datasets, we performed an additional experiment in which we included datasets corresponding to tissues from the following types: adipose, blood, brain, breast, kidney, liver, lung, and stomach. For each tissue type, we gathered two datasets. Here, we wished to elucidate the shared structure across tissue-types, e.g., if it were possible to use *CONFINED* to cluster datasets based on their tissue type. For each pair of datasets, we saved the correlations output by *CONFINED* (i.e., the correlations between the canonical variables as defined in the “Methods” section), and used a statistic of these correlations to construct a distance matrix for use in hierarchical clustering. We took the mean of the top 10 correlations between each pair of datasets, *i,j*, and populated each entry of the matrix _*ij*_ with this mean correlation. Intuitively, this acts a metric of similarity between each dataset. After running hierarchical clustering, we found that tissues of the same type clustered together for each of the datasets (Fig. 6). We believe that this presents evidence that *CONFINED* is in fact finding signal that recapitulates the underlying biology shared between two datasets.

## Discussion

Here, we propose *CONFINED*, a sparse-CCA-based method to capture biologically replicable signal by leveraging shared structure between datasets. Though *CONFINED* captures the shared variability between two datasets, there may be sources of variability that are unknown or unmeasurable present in the datasets, and we cannot evalaute *CONFINED*’s performance for these sources of variability. Therefore, we have highlighted the strength of *CONFINED* through examples of known measured and unmeasured sources of variability. Specifically, we showed its use and improved accuracy over other methods in the context of capturing cell-type composition between datasets of the same tissue type. We also showed how it can be used to capture other sources of biological signal shared across datasets. Moreover, we provide evidence that *CONFINED* can be used as a feature selection mechanism, prioritizing features that are functionally shared between datasets.

Across several datasets we demonstrated that *CONFINED* accurately captured global biological sources of variability. In the case of cell-composition, the components produced by *CONFINED* better captured cell-type composition across all cell-types in methylation datasets (of the same tissue-type) than previous reference-free methods that were designed for capturing signal from cell-type composition. Additionally, *CONFINED*’s components captured other replicable sources of variability such as age and sex. While cell-type composition was better captured when using a pair of datasets of the same tissue-type, we note that other biological factors may be better captured when pairing two datasets of different tissue types. Our results provide grounds for *CONFINED* as a means to capture replicable signal from biological sources across datasets.

Additionally, *CONFINED* is robust to technical variability. Through simulations, we demonstrated that *CONFINED* accurately captures biological signal in the presence of strong, dataset-specific technical noise. Other methods that leverage decompositions of single matrices produced components corresponding to the simulated technical noise (Additional file 1: Figure S13), but the components produced by *CONFINED* were unaffected by the simulated noise. Therefore, leveraging *multiple* datasets through *CONFINED* can provide researchers a way to robustly account for signal arising from technical variation. Though the premise of *CONFINED* is to leverage the shared structure across two datasets to distinguish technical noise, we show in the Supplementary the context in which *CONFINED* uses a single dataset split into halves as input instead of two separate datasets. In this experiment, *CONFINED* suffers from issues similar to single-matrix methods, and its performance was negatively affected by the presence of dataset-specific variability (Additional file 1: Figure S15).

Though we learned a linear rule for selecting the sparsity parameter (i.e., the number of features) in the specific case of capturing cell-type composition in methylation whole-blood datasets (Additional file 1: Figure S10), we emphasize that the selection of the sparsity parameter in other cases may be non-trivial. Evaluating *CONFINED* on multiple datasets and sources of biological variability aside from cell-type composition, we found that the optimal sparsity parameter for cell-type composition may not be optimal for other covariates of interest. For instance, with a pair of blood datasets where the sex chromosomes were removed, sex was better captured as the number of features increased. This may be due to the fact that specific biological functions—such as the immune response—may be confined to several thousand methylation sites, whereas autosomal changes in methylation patterns due to more broad characteristics—such as age or sex—are more minute, and thus require more information or sites to capture. Nonetheless, in Additional file 1: Figure S1 we show that when the sex chromosomes are included in the analysis, the accuracy of *CONFINED* can improve dramatically ($R^{2}_{\text {sex}} >.9$). We suggest future investigations take place and considerations about underlying biology be taken into account for selecting the optimal sparsity parameter for biological signal aside from cell-type composition.

We also showed the utility of *CONFINED* as an unbiased way of selecting informative and potentially biologically relevant methylation sites. Intuitively, as CCA finds shared structure between datasets, this structure should be reflective of biological mechanisms that are common to a pair of datasets. In our experiments, *CONFINED* found methylation sites that capture the shared variability across different blood tissues, and this set of sites was significantly enriched for immune function. Similarly, for the brain-blood pair, we observed enrichment for some immune and hematopoietic function, but the enrichment was generally not significant. Thus, our results suggest that our feature-selection method may be useful in highlighting pathways that are similar across two datasets.

A similar concept to *CONFINED* has been previously introduced in the context of single-cell RNA-sequencing by Butler et al. [48]. However, mathematically, the problem Butler et al. solve is different as the number of “individuals” (in their case, cells) in single-cell RNA is much larger than the number of features (genes), whereas in our setting, the number of individuals is much smaller than the number of features (methylation sites). Moreover, we show that a simple application of CCA does not suffice in the case of methylation, and thus *CONFINED* performs feature selection prior to performing CCA. In other words, *CONFINED* utilizes sparsity.

Importantly, determining the input and usage of the output of *CONFINED* is goal-specific. As the assumption of *CONFINED* is that the biological variability in two datasets is shared, we suggest pairing two datasets with similar characteristics, e.g., design protocol or sample collection. In such cases, for any pair of datasets, *CONFINED* can be used to capture variability or model biological factors that are present in both datasets for use in downstream analyses. On the other hand, *CONFINED* can be used as a preprocessing step to make dataset-specific effects more prevalent. In Fig. 5, we show how *CONFINED* can be used to highlight a treatment effect that was present in a subset of individuals in one of the input datasets. Thus, *CONFINED* enables researchers to decide how they wish to model the shared or unshared variability in their datasets.

The parameters of *CONFINED* can be fine-tuned for downstream analyses. In general, we recommend inducing sparsity to capture variability due to specific functions, such as cell-type composition. For more broad characteristics, such as age and sex, we recommend less sparsity is induced. There may be tradeoffs when attempting to optimize the correlation of the *CONFINED* components and specific sources of variability, and we suggest from our empirical results using around fifty percent sparsity. We found the correlation threshold to be robust across a large range of values (Additional file 1: Figure S16), but suggest using a relatively higher correlation such as.95. Lastly, we suggest using a low number (e.g., 6 or 10) of *CONFINED* components as people often do in EWAS with principal components [24, 49].

In summary, our results suggest that *CONFINED* will be a useful tool in capturing effects of biological variability as well as highlighting shared cellular mechanisms across multiple datasets. The components from *CONFINED* can be used in downstream analyses that wish to model only the biological signal of a methylation dataset or to include certain biological signals as confounders in statistical analyses. We suggest future research into the selection of *t*, the number of informative sites to use for recovering signal for specific biological factors, as well as research into which pairs of phenotypes or datasets may be useful in extracting signal for specific biological drivers of variability. We posit that using extensions of CCA which include more than two datasets [36] may be a promising future direction, however as we show in the Supplementary (Additional file 1: Figure S17), this extension may not be entirely trivial.

## Methods

### A brief introduction to canonical correlation analysis

We first explain the general idea of canonical correlation analysis (CCA) [50]. In the simplest terms, CCA attempts to maximize the correlation of two matrices via linear transformations. CCA takes as input two matrices *X*_1_ of dimension *n*×*m*_1_ and *X*_2_ of dimension *n*×*m*_2_ where *n*>*m*_1_ and *m*_2_. In other words, both matrices have the same number of rows but not necessarily the same number of columns. CCA then attempts to find *m*_1_- and *m*_2_-length vectors *a*_1_ and *a*_2_, such that the correlation of *X*_1_*a*_1_ and *X*_2_*a*_2_ is maximized:

1 $$ \underset{a_{1},a_{2}}{\max}\;\; \text{corr}(X_{1}a_{1}, X_{2}a_{2})  $$

To produce *a*_1_ and *a*_2_, we first obtain vectors *b*_1_ and *b*_2_, the eigenvectors corresponding to the largest eigenvalues of the following matrices (where *X*_1_ and *X*_2_ are column-centered):

$$\begin{array}{*{20}l} M_{1} =& \frac{1}{n}^{1/2}\left({X^{T}_{1}}X_{1}\right)^{-1/2} \left({X^{T}_{1}}X_{2}\right) \left({X^{T}_{2}}X_{2}\right)^{-1/2}\\&\left({X^{T}_{2}}X_{1}\right)\left({X^{T}_{1}}X_{1}\right)^{-1/2}\\ M_{2} =& \frac{1}{n}^{1/2}\left({X^{T}_{2}}X_{2}\right)^{-1/2} \left({X^{T}_{2}}X_{1}\right) \left({X^{T}_{1}}X_{1}\right)^{-1/2}\\&\left({X^{T}_{1}}X_{2}\right)\left({X^{T}_{2}}X_{2}\right)^{-1/2}\\ \end{array} $$

The vectors *a*_1_ and *a*_2_ are then obtained from a simple change of basis of *b*_1_ and *b*_2_ respectively:

$$\begin{array}{*{20}l} a_{1} &= \left(\frac{1}{n}{X_{1}^{T}}X_{1}\right)^{-1/2} b_{1}\\ a_{2} &= \left(\frac{1}{n}{X_{2}^{T}}X_{2}\right)^{-1/2} b_{2}\\ \end{array} $$

The products *X*_1_*a*_1_ and *X*_2_*a*_2_ are referred to as the first canonical variables of the input matrices, and we let *u*_1_=*X*_1_*a*_1_ and *u*_2_=*X*_2_*a*_2_. CCA can produce up to min{*m*_1_,*m*_2_} pairs of canonical variables from the remaining eigenvectors, however, the first pair of canonical variables (corresponding to the largest eigenvalue) has the greatest correlation.

When seeking the second and subsequent pairs of canonical variables, one additional restriction is introduced—the new canonical variables must be orthogonal to all the previous ones:

$$\begin{array}{*{20}l} \text{corr}\left(u_{1}^{(i)}, u_{1}^{(j)}\right) = \text{corr}\left(u_{2}^{(i)}, u_{2}^{(j)}\right) = 0 \;\;\; i< j \end{array} $$

Given this constraint, the solution for the *i*^*th*^ pair of canonical variables conveniently follows the same formula as the first pair, only that we substitute the eigenvector corresponding to the *i*^*th*^ largest eigenvalue for the eigenvector corresponding to the largest eigenvalue. We then column-wise concatenate all $u_{i}^{(j)}$ for each dataset to obtain two matrices (*U*_1_ and *U*_2_) of canonical variables of size *n*×min{*m*_1_,*m*_2_}. Simply put, the collection of canonical variables for each dataset is defined as follows:

2 $$ U_{1} = X_{1}A_{1}\;\;U_{2} = X_{2}A_{2}  $$

Where *A*_1_ and *A*_2_ are the eigenvectors of *M*_1_ and *M*_2_ (after change of basis) respectively. The canonical variables are ordered such that their correlation (which is proportional to their corresponding eigenvalue) is in decreasing order:

$$\text{corr}\left(u^{(i)}_{1}, u^{(i)}_{2}\right) > \text{corr}\left(u^{(j)}_{1}, u^{(j)}_{2}\right) \;\;\; i< j $$

Additionally, the canonical variables have the properties that each of their variances equal 1, and the covariance of $u^{(i)}_{1}$ and $u^{(j)}_{1} \left (\text {and}\ u^{(i)}_{2}\ \text {and}\ u^{(j)}_{2}\right)$ is equal to 0 when *i*≠*j*:

$$\frac{1}{n}{U^{T}_{1}}U_{1} = I, \;\frac{1}{n}{U^{T}_{2}}U_{2} = I $$

To reiterate, the basic goal of CCA is to find *a*_1_ and *a*_2_ such that corr(*X*_1_*a*_1_,*X*_2_*a*_2_) is maximized. There are min{*m*_1_,*m*_2_} such vectors for each pair of datasets, yielding min{*m*_1_,*m*_2_} pairs of canonical variables.

### A formal description of *CONFINED*

CCA has been used in genomics in many instances [51–53]. In these cases, the rows correspond to individuals, while the columns correspond to features of genomic measurements. For example, each feature could be the expression of a specific gene in one matrix, and in the other matrix, it could be the genotype allele, i.e., in this case, *X*_1_ corresponds to a gene expression matrix, and *X*_2_ corresponds to a genotype matrix, but both measurements have been taken on the same set of individuals. In *CONFINED*, we transpose the problem. Rather than searching for shared directions between two sets of genomic measurements, we instead search for shared directions of the same type of genomic measurement (in our case, methylation), but across two sets of individuals. Moreover, since we find that in practice many sources of variability in methylation only act on a fraction of the methylation sites in the genome[14, 24], *CONFINED* uses sparsity by limiting the analysis to a fraction of the methylation sites in the genome. We note that our method shares similarities with a recent application of CCA to single-cell expression datasets [48]. However, unlike this method, we search for shared structure across two sets of individuals rather than two sets of cells, and we assume the number of genomic features is larger than the number of individuals (or cells).

Formally, *CONFINED* takes as input two matrices, *X*_1_ with dimension *m*×*n*_1_ and *X*_2_ with dimension *m*×*n*_2_, of *m* measured methylation sites for *n*_1_ and *n*_2_ individuals respectively. In addition, it takes as input a sparsity parameter *t*, a dimensionality parameter *l*, and an output parameter specifying the number of components to generate *k*. To generate its components, *CONFINED* first selects the *t* most informative features then runs CCA on these *t* features:

1. Obtain *U*_1_ and *U*_2_ both of size *m*×min{*n*_1_,*n*_2_} following Eqs. (1) and (2).
2. Construct $\tilde {U}_{1}$ and $\tilde {U}_{2}$ both of dimension *m*×*l* from the first *l* columns of *U*_1_ and *U*_2_ respectively.
3. Generate a low-rank approximation of each dataset:
  3 $$ \tilde{X}_{1} = \tilde{U}_{1}\tilde{U}_{1}^{T}X_{1} \;\;\;\;\;\;\;\; \tilde{X}_{2} = \tilde{U}_{2}\tilde{U}_{2}^{T}X_{2}  $$
4. For each site *j* in dataset *i*, compute a score based on its correlation between itself and its low-rank approximation:
  4 $$ S_{i}^{(j)} = \text{corr}\left(X_{i}^{(j)}, \tilde{X}_{i}^{(j)}\right)  $$
5. Rank the sites with the highest inter-dataset score:
  5 $$ S_{1}^{(j)}+S_{2}^{(j)}  $$
6. Perform CCA using the sites with the top *t* scores, returning *CONFINED* components $X_{1}^{[t]T} U_{1}^{[t]}$ of size *n*_1_×*k* for *X*_1_ and $X_{2}^{[t]T} U_{2}^{[t]}$ of size *n*_2_×*k* for *X*_2_.

We set *l* as the number of pairs of canonical variables with correlation greater than a threshold *λ*, or 1 in the case that no pairs have this correlation. In practice, we set *λ* to.95 and found this threshold using cross-validation (Additional file 1: Figure S10). By finding the sites that are best approximated by a low-rank, correlated transformation, we therefore assume that the sites with the highest scores will be representative of features that are functionally shared (i.e., correlated) between the datasets. This step is analogous to one taken by ReFACTor [24], only that we leverage the *correlated* subspace of the two datasets rather than a *variable* subspace of one dataset (Additional file 1: Sec. S11). Though we emphasize that *CONFINED* can be used for general sources of global biological variation, for the purpose of comparing a single use-case of *CONFINED* to other methods, we empirically fit a rule for selecting the optimal *t* for cell-type composition in whole-blood datasets as a linear function of the number of individuals in *X*_1_ and *X*_2_ (Additional file 1: Figure S10).

*CONFINED* is available as an R package at https://github.com/cozygene/CONFINED [54]. The calculations in the R package were optimized with C++ code using Rcpp and RcppArmadillo. Also included with the package is an ultra-fast function for performing CCA (Additional file 1: Figure S18).

**Simulations** We evaluated the performance of *CONFINED* using a simulated study. For the simulations, we generated $\widehat {X_{i}}$ for every dataset *X*_*i*_:

$$\widehat{X_{i}} = X_{i} + Z_{i}{W^{T}_{i}} $$

Where *Z*_*i*_ is a random matrix of “scores” of size *m*×*r* with every entry *z*_*jk*_ drawn from the standard normal distribution and *W*_*i*_ is a matrix of “weights” of size *n*_*i*_×*r* where every entry *w*_*jk*_ is drawn from the standard uniform distribution and each column $w^{(k)}_{i}$ is standardized to have norm 1.

In doing so, we add some structured, normally distributed noise that is specific to each dataset. By varying the number and length of the weight vectors $w^{(k)}_{i}$, we can also control the rank and magnitude of the structured noise. Intuitively, this noise emulates technical variation, as each dataset will have its own unique set of weight vectors. For further details, see Additional file 1: Section S7.

### Permutation testing

To validate the enrichment results reported by missMethyl [40], we performed permutation testing. missMethyl takes as input a set (i.e., sample) of CpG sites used to test for enrichment of gene ontology pathways, along with the population from which the sample of CpG sites was chosen. For the purpose of the permutation tests, our sample of CpG sites consisted of the top *t* sites reported by *CONFINED*, and the population of CpG sites was made up of the *m* sites in the input matrices. For each number of sites *t*, we ran missMethyl 1000 times, using a random selection of *t* sites from the *m* sites of the input datasets at each iteration. We then compared the permutation p-values to the p-values from using the top *tCONFINED* sites. For further information, see Additional file 1: Section S6. We also show the results of the permutation test in the presence of noise (Additional file 1: Figure S19)

### Usage of other methods

We compared *CONFINED* against several previous reference-free methods that were developed to capture cell-type composition. Notably, each method has several parameters the user is left to select, and we wished to provide a fair comparison across methods. In the case of PMA[36], we followed the authors’ code and used their cross-validation function to estimate optimal parameters, which balances the fit of the model by optimizing the sparsity. In the case of PEER[41] we simply used the code in the authors’ example in their github wiki. We also followed the authors’ recommendations for optimizing the sparsity parameter and feature-selection steps of ReFACTor[24]. In addition to the above, we also tried each of the methods using the top 1000 to 10,000 most variable sites (with a step size of 1000) for a more fair comparison (similarly to how was done by Houseman et al. [23]). When we induced sparsity in PMA, PEER and NNMF, the methods’ performance were generally lower than when using no sparsity. In terms of *R*^2^, we describe the results when using 10,000 sites and no sparsity respectively: $R_{\text {PMA}}^{2} =.47$ as opposed to.54, $R_{\text {PEER}}^{2} =.49$ compared to.52, $R_{\text {NNMF}}^{2} =.49$ instead of.54. ReFACTor benefited most from sparsity and had the highest performance when using 2000 sites $R^{2}_{\text {ReF}}=.79$.

### Datasets

Throughout our main experiments, we used publicly available data generated from the Illumina Infinium Human Methylation 450k chip. Our analyses focused on four whole-blood datasets and one brain-tissue dataset: (1) an analysis of rheumatoid arthritis patients and controls with 659 individuals from Liu et al. (GSE42861) [39], (2) a study of aging with 656 individuals from Hannum et al. (GSE40279) [38], (3, 4) analysis and re-analysis of schizophrenia with 847 and 675 samples from Hannon et al. (GSE80417, GSE84727) [55], and (5) a dataset from Lunnon et al. with brain tissue from 122 individuals that was used to study Alzheimer’s disease (GSE59685) [43].

The whole-blood datasets were preprocessed following guidelines suggested by Lehne et al. [56]. Using the R package minfi [57], we obtained and subsequently preprocessed the raw IDAT methylation files from the Liu et al. and Hannon et al. datasets. As there was no supplied IDAT file for the dataset of Hannum et al., we simply used their published intensity values. Following the guidelines of Lehne et al., we first removed single nucleotide polymorphism markers (total of 65) then applied the Illumina background correction to the obtained intensity values treating autosomal and sex chromosomes separately. We set our p-value detection threshold to 10^−16^ and set the probes whose p-value did not fall below this threshold as having missing values.

Further, we normalized the whole-blood data using quantile normalization of the intensity values, subdivided by probe type, probe sub-type, and color channel. After finalizing the intensity levels, we calculated beta-normalized methylation levels for each probe. Probes that had more than 10% of their values missing were discarded from the datasets, and the remainder of missing values were imputed using R package impute. Additionally, following [27], we used GLINT [58] to remove polymorphic and cross-reactive sites [59] as well as sites from non-autosomal chromosomes.

The brain dataset from Lunnon et al. was already preprocessed using the function *dasen* from R package wateRmelon [60]. Notably, this function also operates on the raw intensity to generate normalized beta values and uses similar preprocessing steps, including quantile normalization and the removal of single nucleotide polymorphisms. As *CONFINED* takes as input matrices with the intersection of CpG sites in two datasets, the brain dataset was also analyzed with the removal of polymorphic and cross-reactive sites as well as sites from non-autosomal chromosomes.

Additionally, we removed from our analyses outliers and samples with missing information about their sources of variability. Samples whose principal components scores were over four standard deviations away from the mean were excluded, which led to us removing six samples from the Hannum et al. dataset and two samples from the Liu et al. dataset.

We also followed filtering procedures from other works that also used the same datasets, including the removal of consistently methylated or unmethylated sites [24, 27]. Prior to running any analyses, we filtered out methylation sites with standard deviation less than.02. After all preprocessing steps the dataset from (1) Liu et al. had 376021 sites and 658 individuals, (2) Hannum et al. had 382158 sites and 650 individuals, (3) Hannon et al. 381338 sites and 638 individuals, (4) Hannon et al. 382158 sites and 665 individuals, and (5) Lunnon et al. 485577 sites and 451 individuals.

In the analysis across tissue types as well as the brain and adipose analyses in the supplementary sections, we used the respective authors’ preprocessed datasets. Notably, in many datasets, there were multiple studied phenotypes. When available, we used only the healthy individuals for the clustering experiment. We also removed sites with low standard deviation (<.02) as well as sites with missing values. In the Huang et al. stomach dataset [61], the authors processed the raw signal intensities to functionally normalized beta values using minfi, and after filtering missing and low variables CpG sites, there were 304163 sites for 61 individuals. Woo et al. [62] used minfi to generate functionally normalized M-values from stomach mucosa which we transformed to beta values for 42 individuals and 267858 sites. The normalized beta values of the lung dataset from Wielscher et al. [63] were generated using packages from Bioconductor and after our filtering contained 302023 sites measured for 33 individuals. Shi et al. [64] generated their beta values using the R package methylumi to perform exponential background correction and control-probed-based normalization, and after our filtering we were left with 316992 sites for 244 individuals. The brain [65] and liver [66] datasets of Horvath et al. contained Beta MIxture Quantile dilation (BMIQ) normalized [67] beta values for 260 individuals at 315050 sites and 79 individuals at 346808 sites respectively. The adipose and liver datasets from Bonder et al. [68] consisted of Subset-quantile Within Array Normalization (SWAN)-normalized beta values that were preprocessed using the minfi package, and after our filtering, the first adipose dataset had 287438 for 71 individuals, the second adipose dataset had 293425 sites for 71 individuals, and the liver dataset had 265523 for 110 individuals. The kidney dataset of Wei et al. [69] was processed by the R package RnBeads to conduct BMIQ normalization and background correction on their beta values, and after filtering out unhealthy individuals and sites with missing values and low standard deviation, we were left with 89763 sites for 46 individuals. The beta values for the kidney dataset of Ko et al. [70] were processed using Illumina GenomeStudio Software 2011.1 Methylation Module 1.8, and after filtering contained 338312 sites measured at 85 individuals. Teschendorff et al. [71] generated their breast dataset beta values using the minfi R package as well as their BMIQ normalization, and after our filtering, it contained 353644 for 92 individuals. The breast dataset of Song et al. [72] contained after filterting beta values for 121 individuals at 324431 sites and was generated using Partek Genomics Suite and SWAN normalization.

## Additional files

Additional file 1 Contains supplementary methods and information as well as corresponding figures and tables. (PDF 1669 kb)

Additional file 2 Review history contains the pertinent revision information. (DOCX 27.4 kb)

## Data Availability

All datasets analyzed in this manuscript are publicly available. Gene Expression Omnibus (GEO) accession numbers are as follows: [39] GSE42861; [38] GSE40279; [55] GSE80417, GSE847272; [43] GSE59685; [61] GSE103186; [62] GSE99553; [63] GSE63704; [64] GSE52401; [65] GSE64509; [66] GSE61258; [68] GSE61446, GSE61450, GSE61453; [69] GSE61441; [70] GSE50874; [71] GSE69914; [72] GSE101961; [73] GSE74193. *CONFINED* is available under GNU General Public License 3.0 at Github https://github.com/cozygene/CONFINED [54] as well as Zenodo DOI:10.5281/zenodo.3246640 [74].
