# Supplementary material for: CONFINED: distinguishing biological from technical sources of variation by leveraging multiple methylation datasets
Source: Genome Biol. 2019 Jul 12;20:138. doi: 10.1186/s13059-019-1743-y (PMC6624895; doi:10.1186/s13059-019-1743-y)
Supplement: Supplementary file 1 — Contains supplementary methods and information as well as corresponding figures and tables. (PDF 1669 kb) [file 13059_2019_1743_MOESM1_ESM.pdf]

Supplementary Methods and Information

S1 *CONFINED* and measured sources of variability in additional tissue types

We also analyzed the ability of *CONFINED* when looking at two additional pairs of datasets, one composed of two brain datasets (Horvath et al. [65], Jaffe et al. [73]), and another composed of two adipose datasets (Bonder at al. [68]). Included in the adipose datasets were participants' age, sex, and BMI, and in the brain datasets, the participants' age and sex were included. We also show that by including the sites measured on the sex chromosomes, the accuracy of *CONFINED* to capture sex increases dramatically (Figure S1, Figure S2).

S2 Comparison of a PCA-based method and *CONFINED* using *CONFINED* -selected features

Considering that *CONFINED* may better capture cell composition than single-matrix decompositions as it looks at characteristics shared between datasets, we provide a direct comparison of the accuracy of the top-performing single-matrix decomposition method for capturing cell-type composition, ReFACTor, and *CONFINED* using the same feature selection for both methods. We generated the rankings of features as detailed in the feature selection subsection. Even when ReFACTor uses the features generated by *CONFINED*, it captured cell-composition with lower correlation than *CONFINED* (Figure S7).

S3 Single matrix decomposition on the union of two matrices

In this analysis, we evaluated the best-performing single-matrix method for capturing cell-type composition—ReFACTor—against *CONFINED*, using the union of the two input matrices to *CONFINED* as the input for the ReFACTor. To do this, we simply column-wise concatenated the individuals from one dataset to the other, so that the dimension of the new input matrix was $m \times (n_1 + n_2)$. We ran ReFACTor while also supplying it with a covariate as the `covfile` argument a vector that indicated from which dataset the individual originated (e.g. 0 for dataset_1 and 1 for dataset_2). We ran *CONFINED* using the learned rule for selecting the sparsity parameter since the experiment was evaluating each method's ability to capture cell-type accuracy. Notably, in this analysis, using ReFACTor on the concatenated datasets is analogous to *CONFINED*, only that the basis of ReFACTor is PCA, whereas the basis of *CONFINED* is CCA. In both methods, a feature-selection step based on either PCA or CCA is performed on the entirety of the input matrices, which is then followed by performing PCA or CCA on the selected features to generate the components of interest.

S4 Measured sources of variability across methods

In this section, we compare the performance of *CONFINED* to previous methods designed to capture cell-type composition in methylation datasets. Notably, the aim of several of the previous methods (ReFACTor, NNMF) is simply to capture cell-type composition accuracy, and they do not have an emphasis on other global sources of variation such as age, sex, or environment. For *CONFINED*, we used as input datasets from Hannum et al. [38] and Liu et al. [39]. All

other single-matrix decomposition methods used just the dataset from Liu et al. as input. We used 10 components from each method to predict each source of variability in the Liu et al. [39] dataset. *CONFINED* greatly outperformed other methods when capturing both age and sex and performed best for these factors using a large number of features (i.e. low sparsity). This may be an indicator that changes in observed methylation signal due to age or sex are rather subtle, and require many sites to capture. Interestingly, when *CONFINED* used 5000 sites, age was captured with much greater accuracy than sex.

S5 Enrichment Analysis

In this section, we extend the enrichment analysis presented in the main text. We first verified that *CONFINED* selects for immune-related CpG sites in a separate pair of whole-blood datasets (GSE80417 and GSE84727 of Hannon et al. [55], Figure S1). Notably, neither of the Hannon datasets studied a healthy phenotype whereas in the other pair of datasets we used a dataset studying aging. The immune-related pathways were the most greatly enriched pathways when using *CONFINED*'s feature selection for this pair of datasets, however, they were less significantly enriched than in the analysis of the datasets of Liu et al. and Hannum et al.. We also examined the enrichment in a brain-brain pair of datasets (Horvath et al. [65], Jaffe et al. [73]) as well as an adipose-adipose pair of datasets (Bonder et al. [68]). For these two tissue types, we saw enrichment for myelin, neuron and axon-related functions (Table S3) as well as angiogenic functions (Table S2) respectively. The enrichments recapitulated tissue-related functions, however they were not statistically significant using our permutation testing.

We also performed enrichment analysis when there was simulated technical variation added to the more variable probes in the datasets. In this experiment, we added noise to the sites whose standard deviation was in the top p^{th} percent of standard deviations, where we varied p from 1 to 50. For each site, we added normal noise with mean zero and standard deviation equal to the square of the observed standard deviation of the probe. We show the results for the enrichment when pairing two whole-blood datasets (Liu et al. [39], Hannum et al. [38]) and using $t = 2072$ (following the rule we learned from cross-validation and as we did in Table 1 of the manuscript). Notably, the p-value of the enrichment for the top three sites was relatively consistent, and we continued to observe enrichment for immune-related functions (Figure S19).

S6 Cross validation for cell-type composition

Notably, our method has two hyper-parameters, t the number of sites to include, and l the rank of the transformation used to obtain the informative sites. In this section, we explain how we chose values for both of the hyper-parameters. As a reminder, we pick the sites most correlated between their original data matrix and their low-rank approximation, e.g. X_i and $\tilde{X}_i$ (where $\tilde{X}_i = \tilde{U}_i\tilde{U}_i^T X_i$). We will first explain how we choose l.

i Perform CCA on the input matrices

ii Define λ to be some threshold $[0, 1]$

iii Set l to the number of canonical variables of U_1 and U_2 that have correlation $\geq \lambda$, i.e. the number of pairs of columns of U_1 and U_2 whose correlation is greater than or equal to λ

iv If no canonical variables have correlation $\geq \lambda$, set $l = 1$.

We next detail our cross-validation process. We will assume we have l and a ranked list of our features:

1 First, we store a partition of the data for validation purposes.

  i Hold out one third of the sites of each matrix, storing $X_{1\texttt{validate}}$ and $X_{2\texttt{validate}}$, size $\frac{m}{3} \times n_1$ and $\frac{m}{3} \times n_2$ respectively

2 Using the remaining two thirds of the data, $X_{1\frac{2}{3}}$ and $X_{2\frac{2}{3}}$, we will perform training and testing procedures.

  i Randomly split the input matrices $X_{1\frac{2}{3}}$ and $X_{2\frac{2}{3}}$ into two halves: $X_{1\texttt{train}}$, and $X_{1\texttt{test}}$, and $X_{2\texttt{train}}$, and $X_{2\texttt{test}}$ such that each matrix has $\frac{m}{3}$ sites and their corresponding n_i individuals.

  ii On the `train` partitions of the data, run *CONFINED* using the top t features to obtain $A_{1\texttt{train}}$ and $A_{2\texttt{train}}$, the canonical loadings for $X_{1\texttt{train}}$ and $X_{2\texttt{train}}$ respectively.

  iii Find the top t sites of the `test` data partitions and subset the `test` data partitions to size $t \times n_i$ where n_i is the number of individuals in that dataset.

  iv Using $A_{1\texttt{train}}$ and $A_{2\texttt{train}}$, obtain the $t \times n_i$ canonical variables $U_{1\texttt{test}}$ and $U_{2\texttt{test}}$: $X_{1\texttt{test}}A_{1\texttt{train}}$ and $X_{2\texttt{test}}A_{2\texttt{train}}$ respectively.

  v Use $X^T_{1\texttt{test}}U_{1\texttt{test}}$ and $X^T_{2\texttt{test}}U_{2\texttt{test}}$ to predict cell-type composition for each individual in the `test` partition of the dataset.

3 After learning the optimal parameters t^* and l^*, perform our method on the `validation` partition of the datasets.

In this setting, we essentially learn the axes of the most correlated space for the sites of the `train` datasets, and then leverage this space on the `test` datasets to estimate cell-type composition. The canonical weights used for each `test` partition were obtained without using data from any of the samples in the `test` partitions.

We performed our method on `train` and `test` partitions of the data (**2** above) while varying both the value of t and the threshold λ. For each combination of t and λ, we randomly split the data 10 times, then took the average of the R^2 value when using the first 10 components to capture cell-type composition as a metric of accuracy. Regressing t of the best performing set of hyperparameters onto the number of individuals in the datasets, we learned a rule for selecting t in the case of predicting cell-composition in a pair of whole-blood datasets.

When performing the cross-validation of *CONFINED*, we also recorded the runtime as a function of the number of individuals in the datasets. For 100 to 500 individuals (with a step size of 100) in both datasets, the average runtime of *CONFINED* across ten iterations was 35.35, 45.18, 54.44, 73.94, 118.89 seconds respectively. The bottleneck of the computation is a result of calculating the correlation between the input matrices' CpG sites and their low-rank approximations (Methods section) as this operation is typically done on several hundred thousands of sites. The value of λ generally will not affect run-time, as typically < 10 CCA components will have a high correlation. We show the runtime of our CCA implementation in Figure S18 and note that the runtime of *CONFINED* is dominated by the first performance of CCA as t will typically be smaller than the number of sites in the input datasets. We tested *CONFINED* on our cluster (Linux CentOS 6.10) using a single node and R 3.3.3.

S7 Permutation testing

To validate the enrichment results reported by `missMethyl`, we performed permutation testing. `missMethyl` takes as input a set (i.e. sample) of CpG sites used to test for enrichment of gene ontology pathways, along with the population from which the sample of CpG sites was chosen. For the purpose of the permutation tests, our sample of CpG sites consisted of the top t sites reported by *CONFINED*, and the population of CpG sites was made up of the m sites in the input matrices. In this context, we varied t, the number of features to use, and compared the enrichment p-values when using the top t features sorted by *CONFINED* and t randomly selected features. We specifically compare the p-values of the top three most enriched pathways when using t *CONFINED* sites and the single most enriched pathway when using t randomly selected sites from the size m CpG population. The number of features we tested ranged from 1000 to 10000 with a step size of 1000, and we performed 1000 permutations at each number of features. In this experiment, we focused on a blood-blood pair of datasets (Liu et al. [39], Hannum et al. [38]).

S8 Batch effect simulations

Consider one model of principal components analysis:

$$X = ZW^T + \epsilon$$

Where X is a data matrix of size $n \times p$, W is an $p \times k$ matrix containing the k principal components of X (eigenvectors of the covariance matrix of X), Z is a $n \times k$ matrix of scores for each principal component, and ϵ is an n-length vector containing noise. Intuitively, by finding the eigenvectors corresponding to the top k eigenvalues of the covariance matrix of X, we are finding the directions that explain the most variance in the data. While we might expect that cell counts or some other phenotype might be driving the variance of methylation data, variance in biological data is often confounded by different measurement protocols or human error—in other words, batch effects [9]. Therefore, the top k directions of variance in a dataset may correspond to batch effects, or the observed variance in the data may simply by due to different protocols used to produce the data. For our simulations, we generated noise for each dataset X_i following the previously described structure:

$$\widehat{X_i} = X_i + Z_i W_i^T$$

Where Z_i is a random matrix of "scores" of size $m \times r$ with every entry z_{jk} drawn from the standard normal distribution and W_i is a matrix of "weights" of size $n_i \times r$ where every entry w_{jk} is drawn from the standard uniform distribution and each column $w_i^{(k)}$ is standardized to have norm 1.

In doing so, we add some structured, normally distributed noise that is specific to each dataset. By varying the number and length of the weight vectors $w_i^{(k)}$, we can also control the rank and magnitude of the structured noise. Intuitively, this noise emulates technical variation, as each dataset will have its own unique set of weight vectors.

To elucidate the consequences of the simulated batch effects on PCA-based methods, we examined the correlation of ReFACTor's components and the simulated weight vectors. Regressing the artificial noise vectors onto the ReFACTor components, we observe high R^2 values.

Additionally, if the noise we introduced had rank k and large strength (norm), it was captured by exactly the top k ReFACTor components. In cases where the norm of the weight vector was relatively low, ReFACTor's components still captured some of the signal corresponding to the batch effects. These results emphasize that single-matrix decomposition methods may produce components whose signal also includes noise from technical variation.

S9 Preprocessing using Removal of Unwanted Variation (RUV)

In these experiments, we compared previous reference-free methods to *CONFINED* after using RUV as a preprocessing step for the previous methods. RUV uses a set of control probes (either known *a priori* or learned empirically) to generate components that are unrelated to the factor or phenotype of interest [2,9]. In this case, we used the dataset of Liu et al. [39] considering as the factor of interest the phenotype they studied, Rheumatoid Arthritis. Prior to running any of the other reference-free methods, we used RUV to remove unwanted variation, or residual technical effects. In the case of simulated technical noise, we supplied RUV with the specific number of batch vectors at each iteration in the experiment. Here, the performance of earlier reference-free methods improved quite dramatically (Figure S14). We also used this preprocessing step for the real data setting the number of unwanted factors $k = 2$, following the default arguments for similar packages in the minfi [57] R package. The performance in this case was not improved as much as in the scenario with simulated technical noise, and *CONFINED* outperformed previous methods (Figure S14). This may be because in attempting to remove unwanted signal and keep the signal related to the phenotype of interest, RUV is perhaps adjusting away variability associated with cell-type composition.

S10 Using *CONFINED* on a single dataset split into halves

In these experiments, we considered the performance of *CONFINED* when using as input two halves of a single dataset (Hannum et al. [38]). Notably, this violates the key assumption of *CONFINED*—the two input matrices are not independent datasets, therefore the technical variability will be shared across both datasets. In this scenario, we posit that our method will perform similarly to previous methods based on decomposition of a single matrix. Here, we randomly divided the dataset into two halves 10 separate times, and took the average performance of each of the splits. We also evaluated *CONFINED* when simulated technical noise was added to highlight that dataset-specific variability will be an issue in this scenario.

S11 Extending *CONFINED* to more than two datasets

Here, we show some exploratory analysis for extended *CONFINED* to operate on more than two datasets. We considered two cases: (1) when we simple column-wise concatenate an additional dataset to one of the datasets in the original pair and (2) using the iterative sparse CCA algorithm from Witten et al. [36]. Using the concatenated data as input to *CONFINED* performed much better than when using separate matrices as input into the sparse multiple CCA implementation. Further, the results using the concatenated datasets with *CONFINED* were consistent to those reported when using the datasets separately with *CONFINED* .

S12 Feature selection

In this section, we compare the feature selection steps taken by *CONFINED* (a type of sparse CCA) and ReFACTor (a type of sparse PCA). Given input matrices of size $m \times n_1$ and $m \times n_2$, or more generally for the single-matrix decomposition case $m \times n$.

1 Our features are selected in the following manner:

- i Obtain U_1 and U_2 both of size $m \times \min\{n_1, n_2\}$ following Equations (1) and (2).
- ii Construct $\tilde{U}_1$ and $\tilde{U}_2$ from the first l columns of U_1 and U_2 respectively.
- iii Generate a low-rank approximation of each dataset:

$$\tilde{X}_1 = \tilde{U}_1 \tilde{U}_1^T X_1 \qquad \tilde{X}_2 = \tilde{U}_2 \tilde{U}_2^T X_2$$

- iv For each site j in dataset i compute a score based on its correlation between itself and its low-rank approximation:

$$S_i^{(j)} = \mathrm{corr}(X_i^{(j)}, \tilde{X}_i^{(j)})$$

- v Rank the sites with the highest inter-dataset score:

$$S_1^{(j)} + S_2^{(j)}$$

- vi Use t sites with the top t scores when performing CCA.

2 ReFACTor selects CpG sites in the following way:

- i Compute the singular value decomposition (SVD) of a matrix X and to obtain V, the left singular vectors of X.
- ii Construct $\tilde{V}$ by taking the first l columns of V.
- iii Construct a low-rank approximation of X:

$$\widetilde{X} = \tilde{V}\tilde{V}^T X$$

- iv Find the sites that are most correlated between the original dataset and the low-rank approximation of the dataset.
- v Use the top t most correlated sites for X when performing PCA.

Notably, the features selected by our method are important to both datasets. In both datasets, the features are well-represented in a low-dimensional, *correlated* subspace. In ReFACTor, the features are selected if they are well-approximated by the first few principal components in one specific dataset's *variable* subspace. Performing feature selection based on a single-matrix decomposition method does not consider that some of the first few principal components in a dataset may be driven by batch effects.

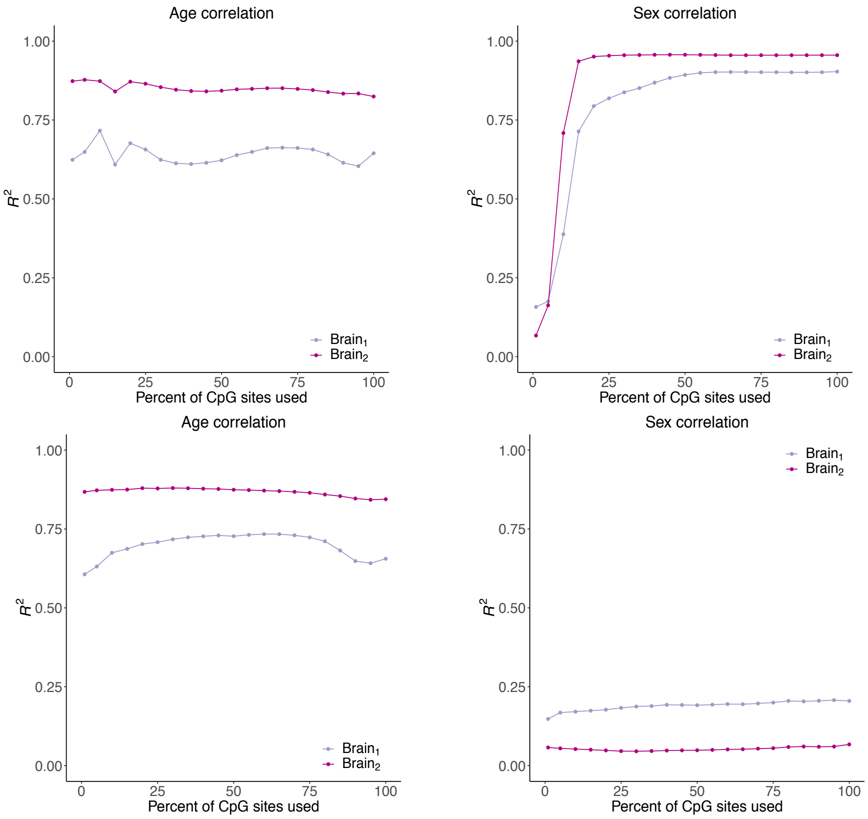

Fig. S1. Evaluation of the ability of *CONFINED* to capture measured sources of variability in brain datasets. We paired a two brain datasets (Horvath et al. [65], Jaffe et al. [73]) to capture sources of variability in each dataset across a range of sparsity. Above, we included the sex chromosomes to show that the accuracy of *CONFINED* to predict biological sex can be improved by the sex chromosomes' site inclusion. Below, we remove the sites along the sex chromosomes.

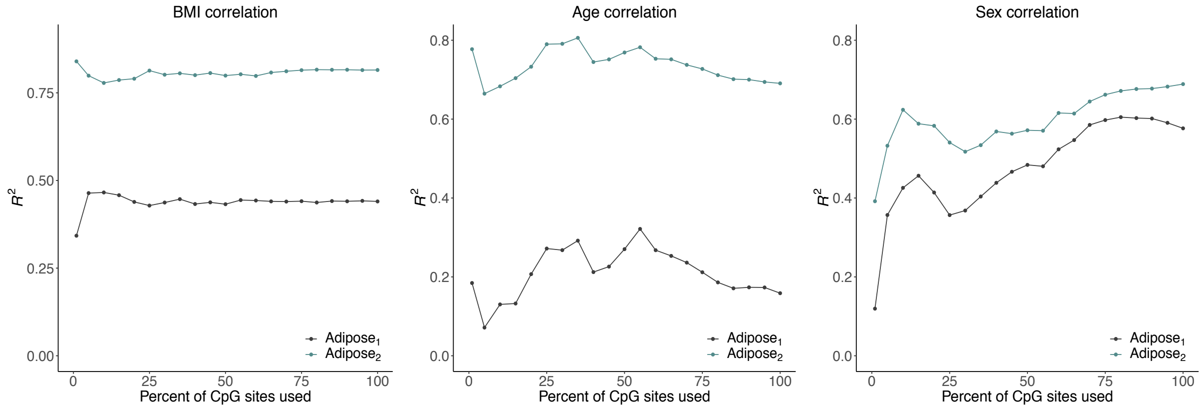

Fig. S2. Evaluation of the ability of *CONFINED* to capture measured sources of variability in adipose datasets. Here, we varied the sparsity parameter for *CONFINED* when capturing body mass index (BMI), age, and sex in two adipose datasets from Bonder at al. [68] using up to 10 *CONFINED* components.

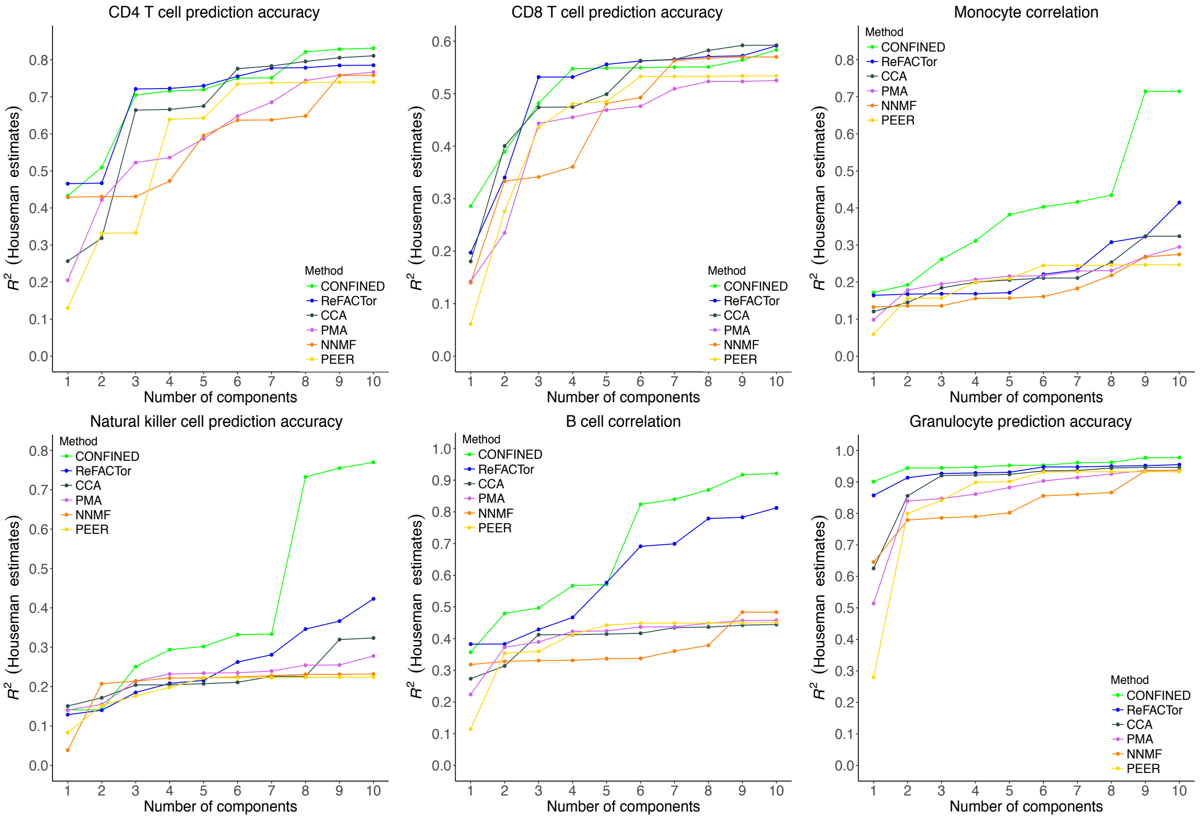

Fig. S3. Evaluation of the ability of *CONFINED* and previous methods to capture cell-type composition. Here, we compare *CONFINED* and previous reference-free methods to capture Houseman estimates for 6 immune cell-types in a whole-blood dataset from Hannum et al. [38] using up to 10 components for each method. For *CONFINED*, we paired the whole-blood dataset from Hannum et al. with the whole-blood dataset of Liu et al. [39].

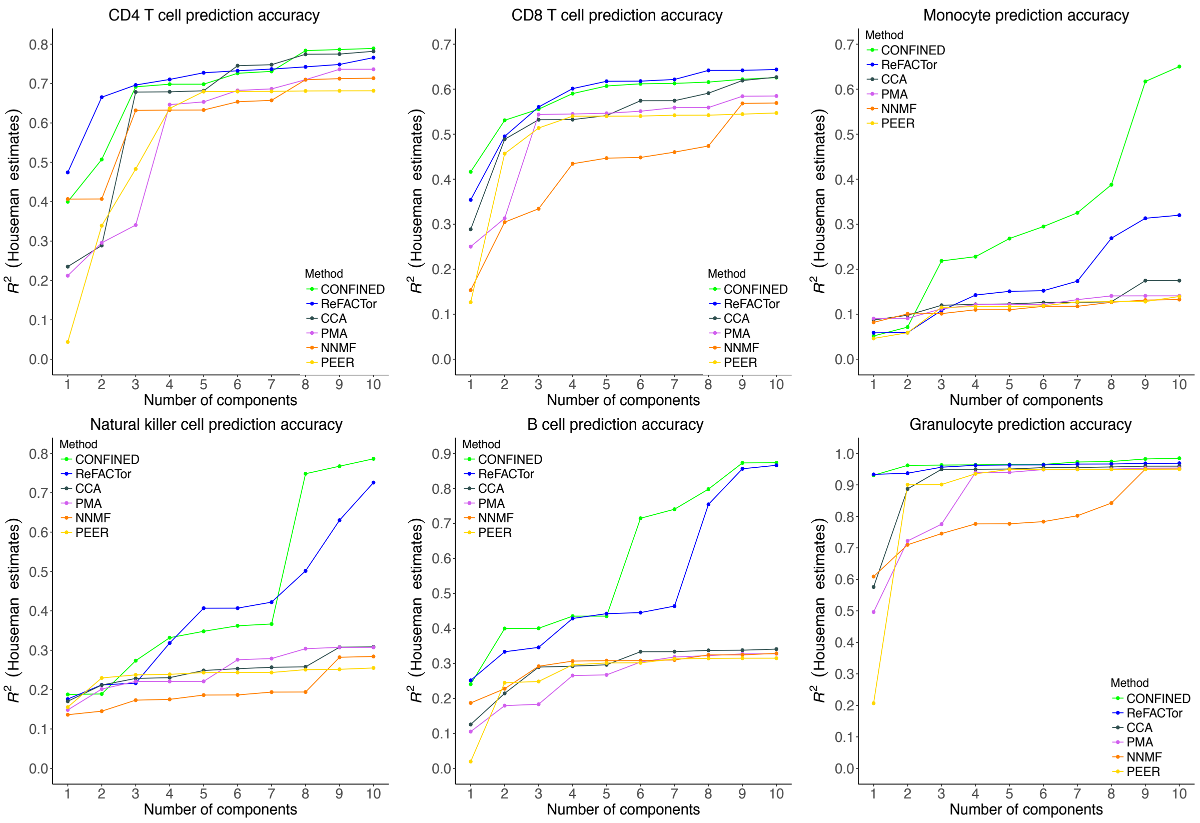

Fig. S4. Evaluation of the ability of *CONFINED* and previous methods to capture cell-type composition. Here, we compare *CONFINED* and previous reference-free methods to capture Houseman estimates for 6 immune cell-types in a whole-blood dataset from Liu et al. [39] using up to 10 components for each method. For *CONFINED*, we paired the whole-blood dataset from Liu et al. with the whole-blood dataset of Hannum et al. [38].

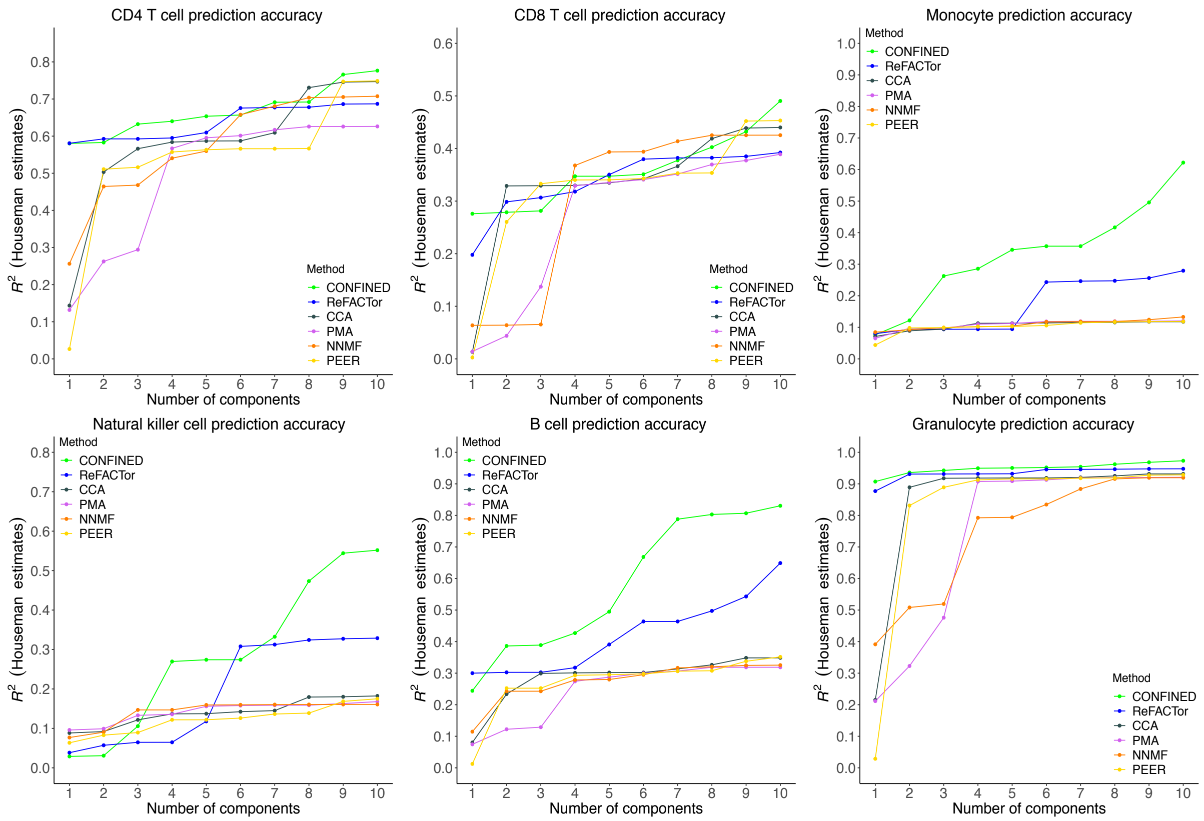

Fig. S5. Evaluation of the ability of *CONFINED* and previous methods to capture cell-type composition. Here, we compare *CONFINED* and previous reference-free methods to capture Houseman estimates for 6 immune cell-types in a whole-blood dataset from Hannon et al. (3, GSE80417) [55] using up to 10 components for each method. For *CONFINED*, we paired this whole-blood dataset from Hannon et al. (3, GSE80417) with the other whole-blood dataset of Hannon et al. (4, GSE84727) [55].

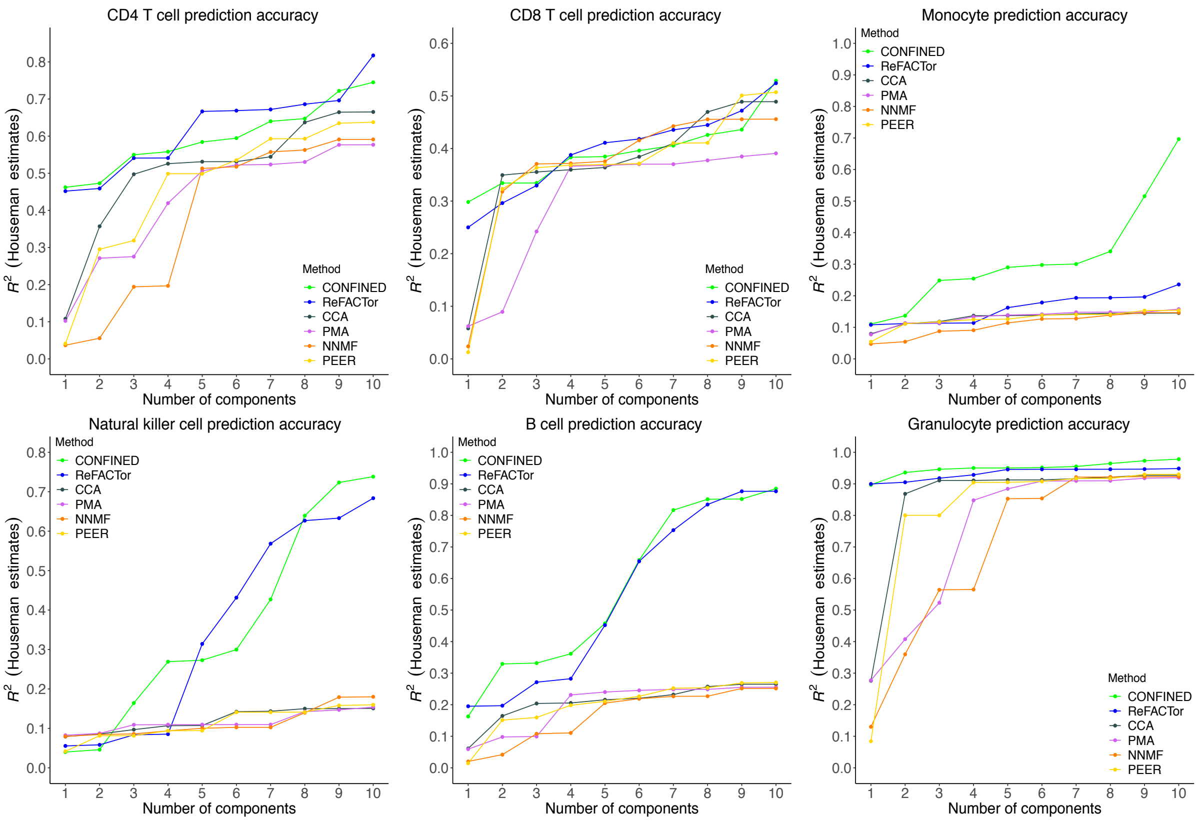

Fig. S6. Evaluation of the ability of *CONFINED* and previous methods to capture cell-type composition. Here, we compare *CONFINED* and previous reference-free methods to capture Houseman estimates for 6 immune cell-types in a whole-blood dataset from Hannon et al. (4, GSE84727) [55] using up to 10 components for each method. For *CONFINED*, we paired this whole-blood dataset from Hannon et al. (4, GSE84727) with the other whole-blood dataset of Hannon et al. (3, GSE80417) [55].

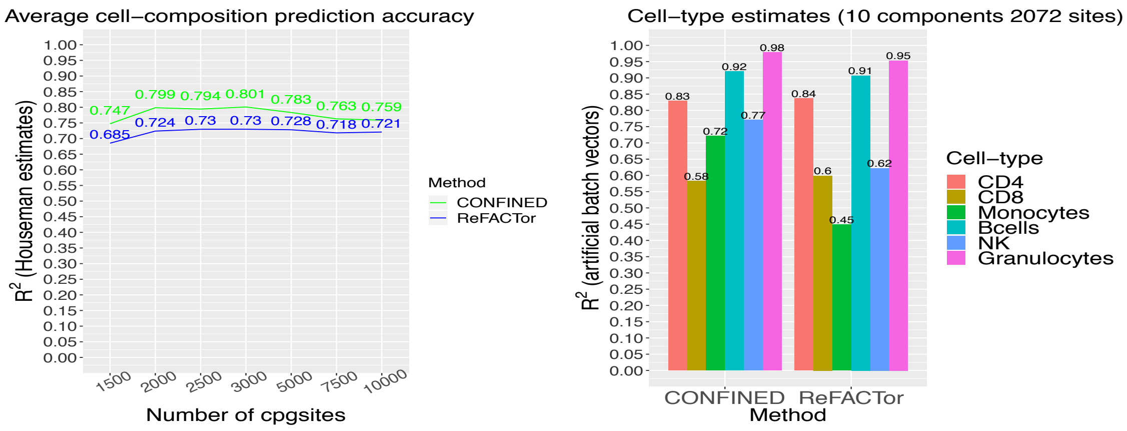

Fig. S7. Comparison of methods based on CCA and PCA. We compared the performance of *CONFINED* and ReFACTor on dataset GSE40279 (Hannum et al. [38]) when both methods used the same features obtained in our feature selection process. On the left, the performance of each method as we varied the number of features. On the right, a comparison of the accuracy of both methods using the the sparsity parameter calculated from cross validation (Additional File 1: Fig. S10).

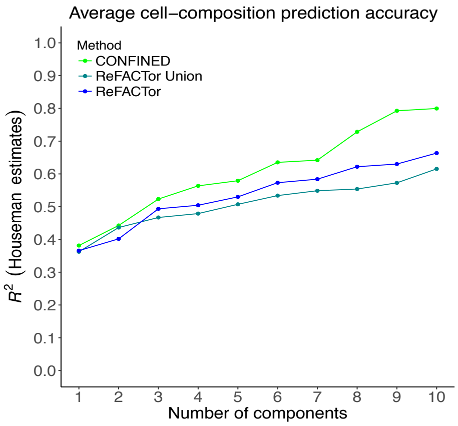

Fig. S8. Single-matrix method using the union of two matrices. In this experiment we used datasets GSE40279 (Hannum et al. [38]) and GSE42861 (Liu et al. [39]). In green, the cell-type composition accuracy of *CONFINED* for dataset GSE40279, in turquoise the accuracy of ReFACTor when using as input the union of the two datasets, and in blue the performance of ReFACTor when just using GSE40279.

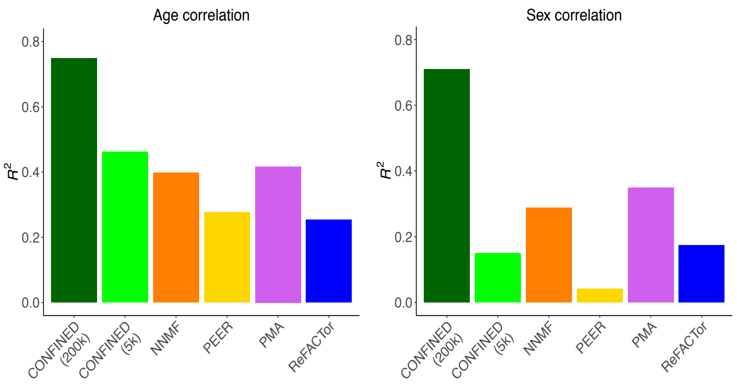

Fig. S9. Correlation of measured sources of global variation with components from *CONFINED* and single-matrix methods. Here, we compare *CONFINED* and previous reference-free methods to capture measured sources of global variation in a whole-blood dataset from Liu et al. [39].

Table S1. Gene Ontology Enrichment of sites ranked by *CONFINED*. We tested enrichment of the highest-ranked sites by *CONFINED* in a blood-blood pair of datasets (GSE80417 and GSE84727, Hannon et al. [55]). Here, we set the sparsity parameter based on a rule learned through cross-validation (Additional File 1: Section S6), however we observed similar results across a range of sparsity parameters (Additional File 1: Section S7).

**Ontology term**	**p-value (permutation)**	**p-value (missMethyl)**
Immune system process	.001	1.1e−13
Immune response	.01	8.18e−12
Cell activation	.04	2.27e−10
Leukocyte activation	.052	3.377e−10
Regulation of immune response	.062	71.16e−10
Protein binding	.071	1.32e−09
Immune effector process	.071	1.35e−09
Regulation of immune system process	.071	1.37e−09
Cytoplasm	.081	2.41e−09
Positive regulation of biological process	.085	2.99e−09
Enzyme binding	.11	8.09e−09
Lymphocyte activation	.11	8.69e−09
Positive regulation of immune response	.12	1.12e−08
Positive regulation of immune system process	.13	2.1e−08
Regulation of leukocyte activation	.14	3.97e−08
Intracellular organelle part	.16	5.54e−08
Response to stress	.16	6.08e−08
Regulation of cell activation	.18	7.11e−08
Neutrophil mediated cytotoxicity	.19	8.73e−08
Positive regulation of nucleobase-containing compound metabolic process	.75	1.46e−04

Table S2. Gene Ontology Enrichment of sites ranked by *CONFINED*. We tested enrichment of the highest-ranked sites by *CONFINED* in an adipose-adipose pair of datasets (Bonder et al. [68]). Here, we set the sparsity parameter to 2000 following the blood-blood enrichment experiments (Table 1, Additional File 1: Figure S11).

**Ontology term**	**p-value (permutation)**	**p-value (missMethyl)**
Blood vessel morphogenesis	.409	3.05e−06
Cardiovascular system development	.422	3.32e−06
Vasculature development	.426	3.49e−06
Blood vessel development	.429	3.57e−06
Angiogenesis	.572	3.63e−05
Regulation of transcription from RNAPII promoter	.618	5.76e−05
Regulation of epithelial cell migration	.619	5.95e−05
Multicellular organism development	.636	6.57e−05
Single-multicellular organism process	.646	6.93e−05
Circulatory system development	.646	7.00e−05
Positive regulation of nitrogen compound metabolic process	.651	7.23e−05
Positive regulation of cellular metabolic process	.658	7.76e−05
Positive regulation of macromolecule metabolic process	.679	8.57e−05
Positive regulation of metabolic process	.691	9.40e−05
Embryo development	.711	1.05e−04
Positive regulation of biological process	.711	1.06e−04
Transcription from RNAPII promoter	.712	1.07e−04
Anatomical structure development	.728	1.23e−04
Epithelial cell migration	.729	1.25e−08
Neutrophil mediated killing of symbiont cell	.19	8.73e−08

Table S3. Gene Ontology Enrichment of sites ranked by *CONFINED*. We tested enrichment of the highest-ranked sites by *CONFINED* in a brain-brain pair of datasets (Horvath et al. [65], Jaffe et al. [73]). Here, we set the sparsity parameter to 2000 following the blood-blood enrichment experiments (Table 1, Additional File 1: Figure S11).

**Ontology term**	**p-value (permutation)**	**p-value (missMethyl)**
Molecular function regulator	.447	2.16e−05
Myelination	.462	2.64e−05
Axon ensheathment	.562	4.50e−05
Ensheathment of neurons	.562	4.50e−05
Positive regulation of hydrolase activity	.655	8.26e−05
Positive regulation of GTPase activity	.659	8.58e−05
Guanyl-nucleotide exchange factor activity	.679	1.04e−04
Ras protein signal transduction	.692	1.11e−04
Voluntary muscoloskeletal movement	.858	2.85e−04
Regulation of GTPase activity	.863	2.96e−04
Regulation of Ras protein signal transduction	.921	4.43e−04
Galactosylceramide metabolic process	.935	5.09e−04
ARF guanyl-nucleotide exchange factor activity	.941	5.46e−04
Negative regulation of peptidyl-serine phosphorylation	.975	8.55e−04
Negative regulation of autophagy	.975	8.56e−04
Regulation of hydrolase activity	.983	9.96e−04
Phosphatase activity	.985	1.05e−03
Negative regulation of endoplasmic reticulum unfolded protein response	.988	1.10e−03
Galactolipid metabolic process	.996	1.36e−03
Ubiquitin conjugating enzyme activity	.996	1.36e−03

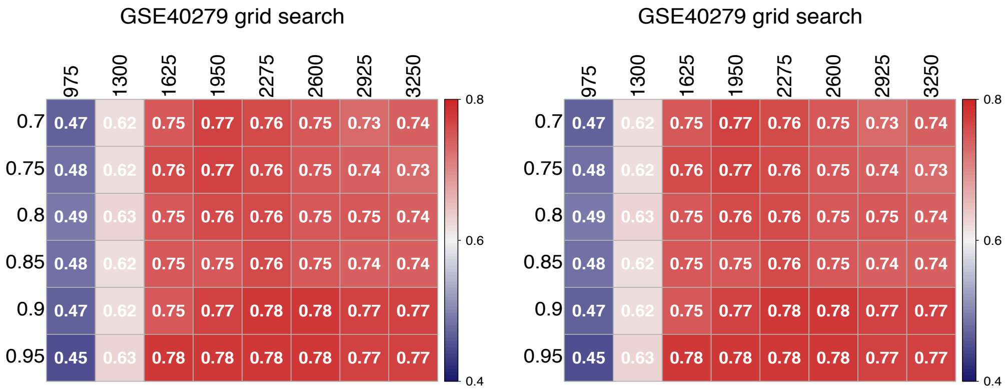

Fig. S10. R^2 **of the testing partitions of the data through varying** t **and** λ**.** We varied the number of sites to use (top axis) and the correlation threshold (side axis) of canonical variables to include in the feature selection step of our method. Each row is a pair of datasets used as input to our method. The number of individuals in datasets GSE40279 (Hannum et al. [38]) and GSE42861 (Liu et al. [39]) was 650 and 658 respectively.

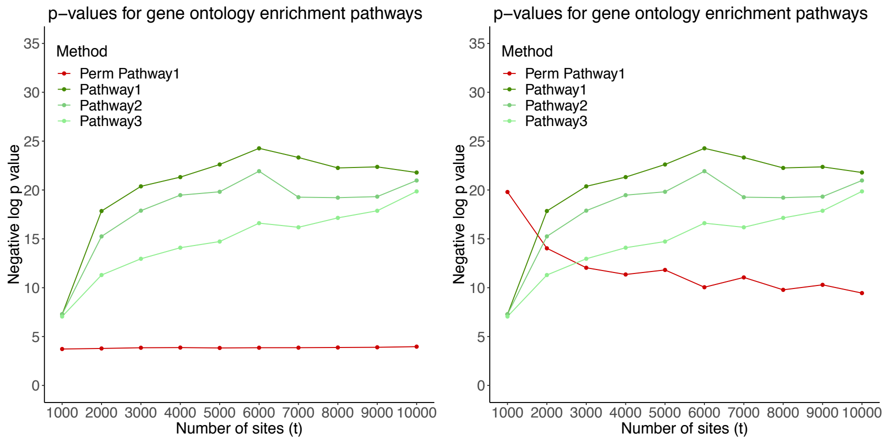

Fig. S11. Permutation testing for gene ontology pathway summary statistics. In green are the top three pathways when choosing the top t *CONFINED*-ordered sites. The red line indicates the average (left) and minimum (right) of 1000 p-values of the top ontology pathway from t randomly selected sites.

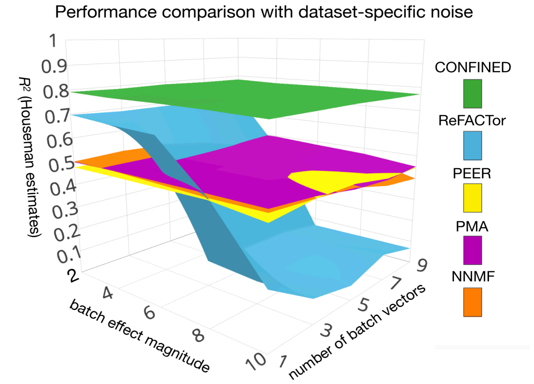

Fig. S12. Capturing cell-composition in the presence of simulated technical noise. We added simulated batch effects to the whole-blood datasets of Liu et al. [39] and Hannum et al. [38] and compared the ability of *CONFINED*, ReFACTor [24], PEER [42], PMA [36], and NNMF to capture cell-type composition in whole-blood. Here, we show the results of the Liu et al. dataset.

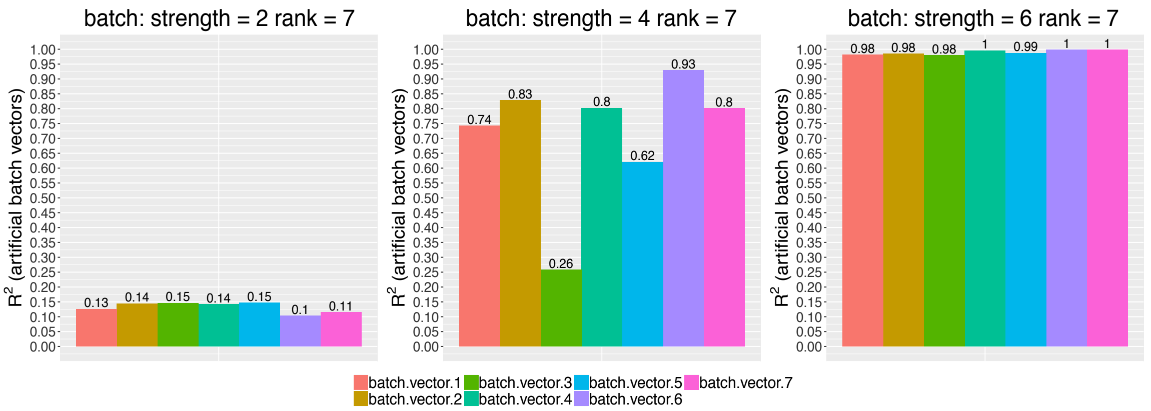

Fig. S13. Batch-effect signal captured by ReFACTor. Here, we examine what portion of the artificial noise is captured by ReFACTor. After adding rank-7-structured noise with different strengths to each dataset, we ran ReFACTor with default settings and examined the correlation between the top 7 ReFACTor components and noise vectors.

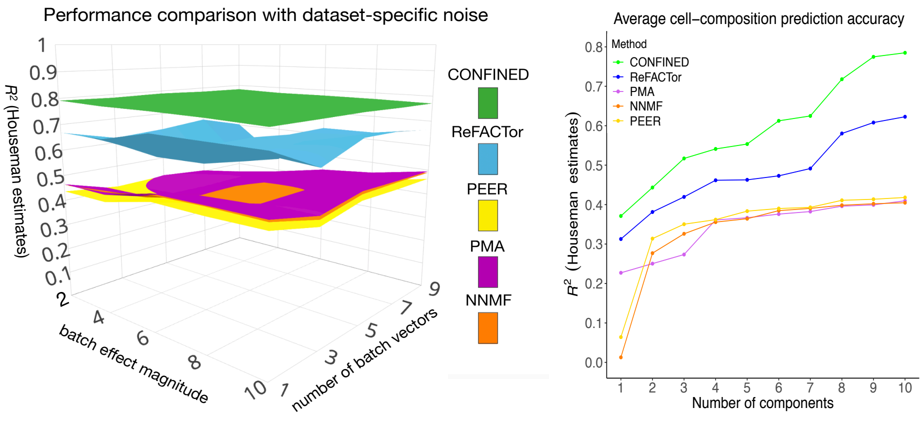

Fig. S14. Using RUV as a preprocessing step to other methods. Here, we compare *CONFINED* and previous reference-free methods to capture cell-type composition in the whole-blood dataset from Liu et al. [39] after using RUV to remove unwanted variation in the datasets. Notably in the case of simulated technical noise (left), these methods improved quite dramatically in comparison to the real data (right).

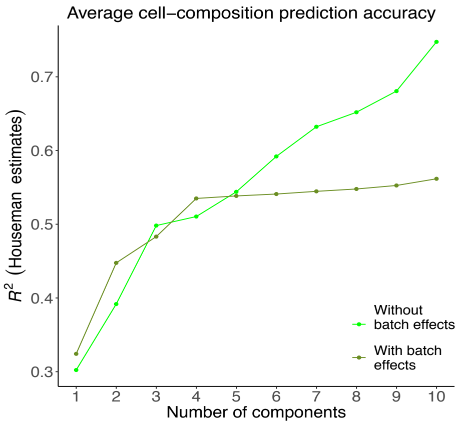

Fig. S15. Using a single dataset with *CONFINED* . In this experiment we split dataset GSE40279 (Hannum et al. [38]) in half and used *CONFINED* to capture cell-type composition estimates as reported by Houseman et al. [19]. We tried this approach on the dataset both with simulated technical noise (dark green) and without simulated technical noise (light green).

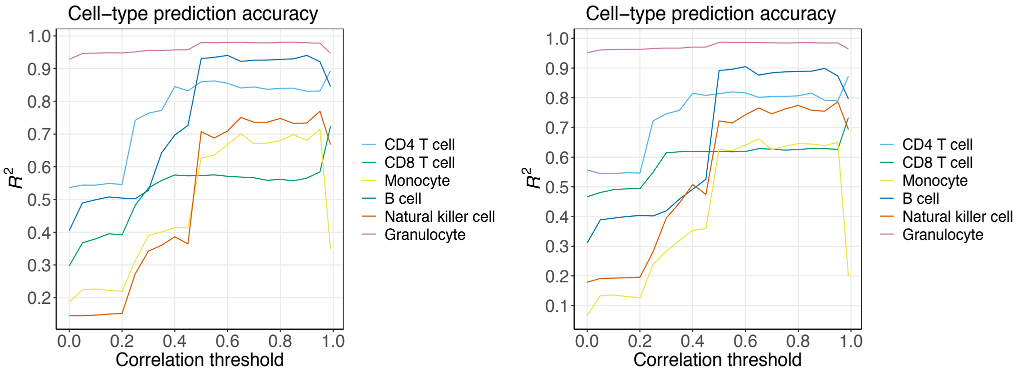

Fig. S16. Average cell-type composition accuracy through varying correlation threshold. Holding the number of sites constant ($t = 2072$, Supplementary Methods; Section S6), we varied the correlation threshold of the feature selection step of *CONFINED* . We show the performance on GSE40279 (Hannum et al. [38], left) and GSE42861 (Liu et al. [39], right) treating cell-type proportion estimates from the algorithm of Houseman et al. [19] as ground-truth.

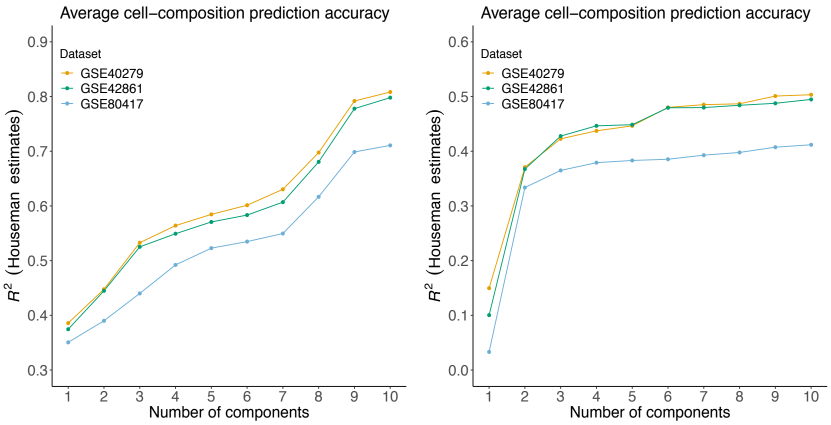

Fig. S17. ***CONFINED* and multiple datasets**. In this experiment we used datasets GSE40279 (Hannum et al. [38]), GSE42861 (Liu et al. [39]), and GSE80417 (Hannon et al. [55]). On the left, when we concatenate GSE40279 and GSE42861 and use the concatenation as input to *CONFINED* along with GSE80417. On the right, when we use all three matrices separately as input to the sparse CCA algorithm of Witten et al. [36].

Matrix entries / Method	10^6	10^7	10^8
CCA::cc	91.85	607.42	5757.08
CONFINED::CCA	35.68	40.17	72.08

Fig. S18. Comparison of CCA implementations. Here we compare the runtime (in seconds) of CCA using the implementation included with *CONFINED* and the CCA package for R.

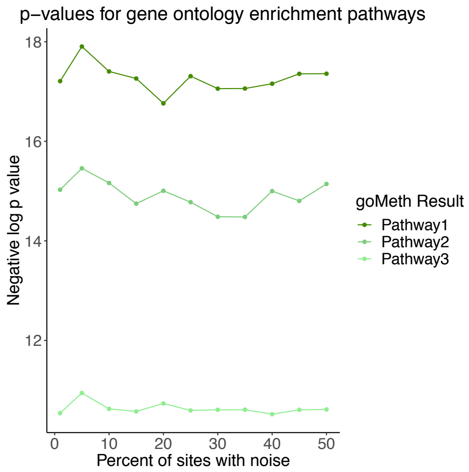

Fig. S19. Enrichment with simulated noise. In this experiment we paired dataset GSE40279 (Hannum et al. [38]) with dataset GSE42861 (Liu et al. [39]) and added noise to the sites with the top highest standard deviation.
