# Supplementary material for: CONFINED: distinguishing biological from technical sources of variation by leveraging multiple methylation datasets
Source: Genome Biol. 2019 Jul 12;20:138. doi: 10.1186/s13059-019-1743-y (PMC6624895; doi:10.1186/s13059-019-1743-y)
Supplement: Supplementary file 2 — Review history contains the pertinent revision information. (DOCX 27.4 kb) [file 13059_2019_1743_MOESM2_ESM.docx]

Review History

**First round of review**

**Reviewer 1**

**Were you able to assess all statistics in the manuscript, including the appropriateness of statistical tests used?**

Yes: No additional review required

**Were you able to directly test the methods?**

No

**Comments to author:**

This paper contains concepts that will have broad relevance. As presented, however, the work feels preliminary.

*	The fundamental assumption is that technical or batch effects will be unique to a dataset, but that biological effects of interest should be common across datasets. There are at least 3 factors that are likely to have effects in most datasets and they are examined in the paper: age, sex and cell type composition. There could be a risk, however, if there are dataset-unique biological factors that dominate. That is, suppose there are factors that have important widespread effects (e.g. age, sex) but that are secondary in magnitude to those that are dataset-unique, such as perhaps a treatment effect. This new method would then adjust away effects of interest. So this would seem to be an extremely important risk.

o	If the authors think that this risk is not important, then they need to defend that position.

o	If the authors acknowledge the risk, then the warning needs to be highlighted and clear in the paper. How should authors choose "two" datasets for this algorithm? Should they restrict to the same or related tissue types? Similar age ranges? Would it be dangerous to have one dataset of men and another of women?

o	Working with one pair of datasets of different tissues (blood and brain) is not enough to convince me that such an analysis is a good strategy in general.

o	Suppose a single dataset were divided randomly into two parts - assuming the dataset was big enough to consider doing so. Could the approach developed in the paper be used to remove batch effects and simultaneously optimize ability to find biological signal? If this were true, then I think the method stands to have more applicability since it would not then rely on the existence of a second appropriate dataset. This could be an exciting potential of the idea. Perhaps multiple random splits could be used and the optimal projections obtained by summarizing across the resulting projections.

*	The method is presented as working with exactly 2 datasets.

o	What kind of comparability would one want to have between the biological effects in the two datasets? I think it would be important to have recommendations on this.

o	Surely the concept could be extended to more than two datasets? Line 257 in the Discussion mentions that this possibility should have interesting consequences, but there are no illustrations. What would be required to implement an augmented method for more than 2 datasets? Could there be at a minimum a discussion of strategies such as performing series of two-dataset analyses versus developing an algorithm explicitly designed to work with more than two datasets? Challenges, opportunities, etc.

*	The methodological details are in a supplement but the main parts of the method should be presented in the main text.

*	I'm not sure of the benefit of having the pathway analysis in the paper. The point seems to be merely that reasonable pathways are identified. Again, as said above, this arises from an "example" analysis of two datasets. However, there are deeper implications that are not explored.

o	What about the role of the number of elements "t" versus the success of pathway analysis?

o	When working with two datasets from blood/brain, identifying immune-related pathways is not surprising. But I think there could be connections with the estimated cell type proportions that open some interesting questions about which probes distinguish between tissues and cell types, and the roles of these probes in distinguishing "technical noise" versus true biological signals. What if the technical have more impact on probes with larger standard deviations in methylation levels across samples? Will performance of the method be affected?

*	The formula on line 803 appears without much motivation or rationale. Does this equation provide any intuition or is it helpful to the paper in any way?

*	The set of comparison methods is missing an important method. The batch correction built into minfi is explicitly designed to remove technical factors and leave biological effects, and yet this method is not included in the comparison.

o	If this method were included, the simulation design for technical factors would need to be redesigned so that the simulated technical noise is correlated with control probe data.

*	Line 268: effects due to sex on methylation cannot be considered minute if one included X and Y, but they were removed here. Discussion required on whether their addition would be helpful.

Minor points:

Figure S6: legend seems inaccurate. "Each row is a pair of datasets"? Why is the blue column placed on the right - essentially out of order w.r.t. the x-axis labelling?

Supplement S7: some formatting issues in the figure legends on the plot. X-axis legend should say (t).

Some comments on the computational time as a function of n_1, n_2, and choices of t would be helpful given that there is a grid evaluation of different values of t and lambda as well as multiple resampling of training/test and cross validation.

**Reviewer 2**

**Were you able to assess all statistics in the manuscript, including the appropriateness of statistical tests used?**

Yes, statistical tests are fine.

**Were you able to directly test the methods?**

No.

**Comments to author:**

The authors present a new method called CONFINED for identifying "hidden factors" representing potential biological confounding factors in DNA methylation (DNAm) data. Identifying such confounding factors is a critical step in EWAS, and typically allows one to tease apart some trivial associations that arise from population biases or cellular heterogeneity. Specifically, the authors posit that by integrating two separate DNAm datasets, one can more readily identify biological sources of variation that are common across populations. Examples of such factors include age, sex, and cellular heterogeneity. Formally, CONFINED is a modified version of Canonical Correlation Analysis (CCA) for finding factors that jointly capture variability across two datasets. The authors show promising results in recovering age, sex, and cellular heterogeneity when the data is further impacted by batch effects.

Overall, the approach sounds interesting and results are promising. However, I think in the current form, the analysis is too superficial on several fronts: 1) the user is yet again left with the main problem of choosing at least 3 hyper-parameters. The current results do not paint a comprehensive picture about relevance and sensitivity of results to these parameters. A more thorough assessment is needed to guide the user, otherwise I don't foresee the method to be of wide usability. 2) Comparison with other methods suffers from the common problem of "lack of optimization" of those other methods (see below). 3) More data is needed to convince the user that CONFINED factors are relevant beyond the three obvious variables (age, sex, and cellular heterogeneity) that are typically easy to recover by existing methods in the absence of noise. I'll elaborate below.

* Comparison to other methods. I found the comparison with existing method unsatisfactory in a typical way: CONFINED has at least 3 parameters that the authors play with, to ensure adequate performance, but it's not clear if the same level of attention was placed in adjusting the parameters of the other methods. For example, PEER has several parameters for balancing fit vs sparsity vs number of factors included.

Related to above, the authors seem to suggest that by using the same number of latent factors, the different approaches are put on similar footing for comparison (e.g., Figure 3). But this is clearly not true: the number of factors mean very different things, e.g., in sparse PCA, 10 PC would explain much fewer amount of variance, compared to 10 PCs in standard PCA. Likewise, whether or not 10 PEER factors have the same "explanatory power" as 10 CONFINED factors depend on sparsity level and other parameters in PEER.

It was not clear to me how the comparison in Figure 2 was done. Specifically, how were the parameters set and optimized?

* Since CONFINED is using two datasets, whereas the other methods compared to use one dataset, it's not possible to distinguish whether the improved performance comes from the method itself or addition of new data. Thus the comparison to PCA applied to concatenated data is key for teasing these two apart. However, I found the comparison in this section superficial and lacking details. For instance, in CONFINED CCA is done twice (once to find t probes (feature selection) and once to construct a final set of latent variables from those selected probes). I suspect that the feature selection step is helping the approach, and if similar procedure was used for the "concatenated PCA", the results would be more comparable.

Minor:

* This statement needs clarification "In these experiments, we considered only known shared biological sources of variation and therefore excluded from our analyses sources of variation that may only appear in one dataset, e.g. patient status or brain location." Do you mean you explicitly modeled the effect of these variables, analogues to what is done in SVA to not over correct?

* I found the Gene Ontology section not that useful. It would be more informative to compare GO analysis between methods, otherwise its hard to say if the enrichment levels are interesting/different or typical in some way.

**Authors Response**

**Point-by-point responses to the reviewers’ comments:**

Reviewer #1: This paper contains concepts that will have broad relevance. As presented, however, the work feels preliminary.

* The fundamental assumption is that technical or batch effects will be unique to a dataset, but that biological effects of interest should be common across datasets. There are at least 3 factors that are likely to have effects in most datasets and they are examined in the paper: age, sex and cell type composition. There could be a risk, however, if there are dataset-unique biological factors that dominate. That is, suppose there are factors that have important widespread effects (e.g. age, sex) but that are secondary in magnitude to those that are dataset-unique, such as perhaps a treatment effect. This new method would then adjust away effects of interest. So this would seem to be an extremely important risk.

o If the authors think that this risk is not important, then they need to defend that position.

o If the authors acknowledge the risk, then the warning needs to be highlighted and clear in the paper.

*We believe that the reviewer’s comment is due to a lack of clarity in our explanation of the objectives of our method, and we therefore now clarify this in the paper as well as below. Specifically, the goal of CONFINED is to find shared variability across datasets. In a typical experiment there are multiple sources of variations:*

*1. Known and measurable sources such as age and sex. These are typically accounted for by adding them explicitly to the statistical model in a downstream analysis.*

*2. Known but unmeasured sources such as cell type composition or ancestry. These are typically accounted for by first estimating them (using supervised or unsupervised methods), and then adding their estimates explicitly to the model.*

*3. Unknown sources. These can be partitioned further into technical and biological factors. Since these sources are unknown, it is impossible to use supervised learning to estimate them, and therefore typically unsupervised methods are used, e.g., PCA, SVA, or PEER. However, these methods do not distinguish between the biological and technical factors. The goal of CONFINED is to detect such unknown sources that appear in more than one dataset. Since technical effects are unlikely to be shared across different datasets, our assumption is that these effects would tend to be biological factors. Due to the above, CONFINED will never pick up dataset-specific effects such as a treatment effect that is only given in one of the datasets. Our original manuscript in fact provided evidence that this is indeed the case; we demonstrate using simulations that batch effects do not affect our method, while they do affect other unsupervised methods such as PCA, PEER, and SVA. While our simulations interpreted the added signal as “technical noise,” the same simulations could have been interpreted as other signals that are unique to a datasets, e.g., treatment effect. If the goal were to elucidate the effects of such a treatment, and one wished to capture and control for covariates, PCA and other methods would fail and adjust away the effect of interest. We now clarify the above distinction and objective of the method in page 4 in the Introduction as well as in page 13 of the Discussion.*

*In addition, as a follow-up on the reviewer’s comment, we thought it would be beneficial to demonstrate how CONFINED can actually be beneficially used to better detect the factors that are unique to a dataset by applying it as a preprocessing step for traditional methods such as PEER, SVA, PCA. In the revised manuscript, we added an experiment that demonstrates this point. Specifically, we added a simulated treatment effect signal to half of the individuals in one of the datasets. We then ran CONFINED on the dataset where half of the individuals had a treatment effect and another dataset with no such added effect. We then regressed out the CONFINED components from the dataset with added treatment effect, and we ran PCA on the resulting data. We compare the PCA plot before and after using CONFINED. Notably, the variability between the control and treatment group remains after using CONFINED.*

*We have added this experiment as well as the above figure (Figure 4 in the revised manuscript) to the Results section (page 9) as well as some commentary to the Discussion section (page 13) of the manuscript to highlight this point to future readers.*

How should authors choose "two" datasets for this algorithm? Should they restrict to the same or related tissue types? Similar age ranges? Would it be dangerous to have one dataset of men and another of women?

*We refer the reviewer to our response for the previous comment. As explained above, there is no risk in choosing two datasets in the sense of “matching” shared factors. CONFINED will always only search for the factors that are common to two datasets. We note that the underlying goal and motivation of CONFINED is to search for unknown biological confounders. Typically, scenarios such as the one described by the reviewer consist of confounders that are known, since the studies were designed using these confounders in mind. Known confounders are easier to deal with since they can be directly introduced to the statistical model in the downstream analysis. Since unknown confounders are unknown, it is impossible to evaluate the method - for this reason, we use sex, age, and cell-type composition, which are known shared confounders, but the premise of this method is that there may be other confounders that are unknown and shared across multiple datasets. Therefore there is absolutely no risk in working with any pair of datasets. We now added text clarifying this point in the Discussion (page 11,13).*

o Working with one pair of datasets of different tissues (blood and brain) is not enough to convince me that such an analysis is a good strategy in general.

*We concur that more evidence may be useful to demonstrate the value of CONFINED. In order to address this comment, we enhanced considerably our set of tissues, and we now included datasets corresponding to tissues from the following types: adipose, blood, brain, breast, kidney, liver, lung, and stomach. For each tissue type, we gathered two datasets. Our results show that shared biological sources are captured well using CONFINED. Particularly, if this is the case, then we expect CONFINED to find more similarities between pairs of datasets of the same tissue, compared to pairs of different tissues. Empirically, we found this to be the case. More specifically, we added the following analysis to the paper. For each pair of datasets, we saved the correlations output by CONFINED, (e.g. the correlations between the canonical variables as defined in the Methods section) and used a statistic of these correlations to construct a distance matrix. We took the mean of the top 10 correlations between each pair of datasets, i,j, and populated each entry of the matrix with this mean correlation. This acts as a metric of similarity between each dataset. We then applied hierarchical clustering to the distance matrix and found that tissues of the same type clustered together for each of the datasets. We believe that this presents evidence that CONFINED is, in fact, finding a signal that recapitulates the underlying biology shared between pairs of datasets. The new experiment and the results are now described in the Results section, page 11, and we have also added Figure 6 to the revised manuscript, which illustrates the tree structure generated by the above procedure. The figure is provided below for your convenience.*

o Suppose a single dataset were divided randomly into two parts - assuming the dataset was big enough to consider doing so. Could the approach developed in the paper be used to remove batch effects and simultaneously optimize ability to find biological signal? If this were true, then I think the method stands to have more applicability since it would not then rely on the existence of a second appropriate dataset. This could be an exciting potential of the idea. Perhaps multiple random splits could be used and the optimal projections obtained by summarizing across the resulting projections.

*We argue that splitting a single dataset to two datasets and then running CONFINED would result in a missed opportunity to capture the biological sources of variability, and that the technical sources may dominate the signal, over the biological sources. A key limitation of previous single-matrix methods is that their components may contain dataset-specific noise that is potentially due to technical effects. Therefore, the premise of our method (as well as its novelty) is in that we utilize two datasets and perform a decomposition that will not generate components containing any dataset-specific noise by searching only for variability that is shared across the two datasets. The issue of using a single dataset therefore negates our premise, as the dataset-specific variability will be inherent in both halves. Thus, performing CONFINED on the two halves will result in the extraction of the technical effect, similar to PCA and other methods, and therefore the entire advantage of the method will be lost. In the revised manuscript, we have performed an analysis of our method when splitting a single dataset into two halves under two scenarios; with and without simulated technical noise. When using the matrix on a dataset split into halves, the method performs worse than when using two separate datasets and is more vulnerable to technical effects (Supplementary Information; Figure S2). We added clarification about the risk in such a strategy in the text (Discussion, page 12).*

* The method is presented as working with exactly 2 datasets.

o What kind of comparability would one want to have between the biological effects in the two datasets? I think it would be important to have recommendations on this.

*Since CONFINED detects the shared sources of variation of any pair of datasets, there is no explicit restriction on using specific pairs of datasets for the analysis with CONFINED. However, different pairs of datasets may provide different insights. For this reason, we suggest pairing two datasets with similar characteristics, e.g. design protocol or sample collection. In such cases, for any pair of datasets, CONFINED can be used to capture variability or model biological factors that are present in both datasets for use in downstream analyses. On the other hand, CONFINED can be used as a preprocessing step to make dataset-specific effects more prevalent, as described above. In the revised version, we have added commentary about this in the Discussion section (page 13).*

o Surely the concept could be extended to more than two datasets? Line 257 in the Discussion mentions that this possibility should have interesting consequences, but there are no illustrations. What would be required to implement an augmented method for more than 2 datasets? Could there be at a minimum a discussion of strategies such as performing series of two-dataset analyses versus developing an algorithm explicitly designed to work with more than two datasets? Challenges, opportunities, etc.

*In the revised manuscript, we explored two types of modifications to the traditional CCA In the first case, we simply concatenated one dataset to one of the original two input datasets (i.e. we had three datasets, but one input matrix to the method was a concatenated pair of datasets). Here, we observed only subtle differences compared to the original algorithm that uses two datasets (Supplementary Methods; Figure S4). We also tried the implementation of sparse CCA proposed by Witten et al. Yet again, the performance of CONFINED did not improve. Nonetheless, we agree with the reviewer that the incorporation of multiple datasets (more than two) is a promising future direction that we hope the community will explore, however, there does not seem to be a trivial extension that provides significantly improved results. Particularly, while there are a few methods for CCA across multiple datasets (e.g., Witten et al. 2009 Biostatistics) they are relatively understudied in comparison to traditional CCA, and it is possible that the lack of improvement that we find is due to the limited effort in the literature to provide improved multi-CCA methods. We have added some commentary about this in the Discussion section (page 13).*

* The methodological details are in a supplement but the main parts of the method should be presented in the main text.

*We have updated the Methods in the main text.*

* I'm not sure of the benefit of having the pathway analysis in the paper. The point seems to be merely that reasonable pathways are identified. Again, as said above, this arises from an "example" analysis of two datasets. However, there are deeper implications that are not explored.

*We thank the reviewer for this comment. The purpose of the pathway analysis was to show that CONFINED is selecting sites that recapitulate the underlying biology that its components are capturing. In the example we showed, the several thousand CONFINED-selected sites used for two whole blood datasets were enriched for immune function. In the revised text (Supplementary Methods; Section S4, page 28), we expanded upon this analysis by testing the enrichment of CpG sites selected by CONFINED using an additional pair of whole-blood datasets, a pair of brain tissue datasets, and a pair of adipose datasets. In each respective analysis, the CONFINED-ranked sites were enriched for immune function, various brain-related functions (e.g. myelin, neuron, and axon functions), and vascular-related functions (e.g. angiogenesis).*

*We believe that this shows that the sites selected by CONFINED capture intuitively relevant biological functions.*

o What about the role of the number of elements "t" versus the success of pathway analysis?

*We thank the reviewer for raising this concern. In the original manuscript, we performed an analysis that can be found in the Supplementary Methods section of the paper (Section S9) titled “Permutation testing”. In this experiment, we evaluated the success of the pathway analysis as the number of features t changes. To highlight the advantage of the sparsity parameter, we limit the analysis to at most 10,000 sites. For two whole blood datasets, there was an enrichment of immune function across values of t. We show results of the top 3 CONFINED-selected pathways’ p-values in comparison to the average of 1000 permutation p-values and minimum of 1000 permutation p-values. We suggest that under sparse conditions, the pathway analysis is robust to the choice of t.*

o When working with two datasets from blood/brain, identifying immune-related pathways is not surprising. But I think there could be connections with the estimated cell type proportions that open some interesting questions about which probes distinguish between tissues and cell types, and the roles of these probes in distinguishing "technical noise" versus true biological signals. What if the technical have more impact on probes with larger standard deviations in methylation levels across samples? Will performance of the method be affected?

*We appreciate the reviewer’s comment and apologize for misconstruing the interpretation of our results. The point of the enrichment was not necessarily to discover and posit that we have shown novel pathway enrichments, but rather to provide evidence that our method is in fact finding the shared biology across two different tissue types. In the case of blood and brain datasets, we agree that identifying pathways related to hematopoiesis and immune function may not be surprising, but it is sound biologically.*

*In the revised manuscript, we demonstrate through an additional experiment that adding noise to the probes with higher standard deviation does not have a large impact on the results of the enrichment analysis. In this experiment, we added noise to the sites’ whose standard deviation was in the top pth percent of standard deviations, where we varied p from 1 to 50. For each site, we added normal noise with mean zero and standard deviation equal to the square of the observed standard deviation of the probe. We show the results for the enrichment when pairing two whole blood datasets and observe that the p-value of the enrichment for the top three sites was relatively consistent, and we continued to observe enrichment for immune-related functions. The description of this experiment is now given in the Supplementary Section of the revised manuscript (page 28).*

* The formula on line 803 appears without much motivation or rationale. Does this equation provide any intuition or is it helpful to the paper in any way?

*We agree with the reviewer that this equation may not be necessary to include as a final result and therefore removed it from the paper.*

* The set of comparison methods is missing an important method. The batch correction built into minfi is explicitly designed to remove technical factors and leave biological effects, and yet this method is not included in the comparison.

*We thank the reviewer for raising this concern. Following the framework of removal of unwanted variation (RUV), Minfi acts to remove technical factors as identified by performing principle components analysis (PCA) on “unrelated” or control probes in a dataset. Therefore, these methods assume that there are control probes or probes uncorrelated with the phenotype of interest. Importantly, Minfi operates on the raw signal intensities of a bisulfite sequencing array. In many cases, it may not be possible to obtain the raw, unprocessed files. In this case, one would need to perform a removal of technical factors using another method. In our experiments, we used the beta values from processed methylation datasets. We therefore used RUVm, a method recently designed for the specific case of processed methylation datasets as a preprocessing to running other methods. When we used RUV for preprocessing, the performance of the methods improved and were generally unaffected by the technical effects, but CONFINED still outperformed the previous methods (Supplementary Methods; Figure S19). In the case of the real data, CONFINED outperformed the previous methods as well. This analysis can now be found in the revised manuscript in the Supplementary Methods section S12 (page 47).*

o If this method were included, the simulation design for technical factors would need to be redesigned so that the simulated technical noise is correlated with control probe data.

*Implicitly, the technical noise is correlated with the control probes that minfi or RUV select. These methods assume that the technical effects are global (i.e. affect all the sites) and will select sites that are unrelated to the phenotype either by design (e.g. control probes) or by some other criterion (e.g. correlation). When we add noise to the entire dataset, we will add noise to all probes, including these control probes. In the Methods section (page 16), we show that the way in which we add the noise leads to control probes to consist of a linear combination of the added noise and their original signal.*

* Line 268: effects due to sex on methylation cannot be considered minute if one included X and Y, but they were removed here. Discussion required on whether their addition would be helpful.

*We agree with the reviewer and apologize that we made this unclear, we have since added some additional commentary to the Discussion section of the manuscript (page 12). Our intention was that the effects on the CpGs contained in autosomal chromosomes due to sex are rather minute, or spread out amongst many sites, in whole-blood or the brain. Including X and Y would make finding the variability associated with sex completely trivial assuming there were a male and female present the two datasets. We show an example of this in the revised Supplementary Methods (section S3 page 26), where we included the sex chromosomes when pairing a pair of two brain tissue datasets, and show that CONFINED can nearly perfectly capture sex with several components. Notably, when including the sites located on the sex chromosomes, we can easily capture sex even under sparse conditions, as opposed to when the sites along the sex chromosomes are removed.*

Minor points:

Figure S6: legend seems inaccurate. "Each row is a pair of datasets"? Why is the blue column placed on the right - essentially out of order w.r.t. the x-axis labelling?

*We have made the caption more clear and fixed the ordering in the x-axis.*

Supplement S7: some formatting issues in the figure legends on the plot. X-axis legend should say (t).

*We have updated the figure axis and legend in the revised manuscript in accordance with your suggestion.*

Some comments on the computational time as a function of n_1, n_2, and choices of t would be helpful given that there is a grid evaluation of different values of t and lambda as well as multiple resampling of training/test and cross validation.

*We have added information about runtime to the cross-validation section in the Supplementary methods (Section S9 page 42).*

Reviewer #2: The authors present a new method called CONFINED for identifying "hidden factors" representing potential biological confounding factors in DNA methylation (DNAm) data. Identifying such confounding factors is a critical step in EWAS, and typically allows one to tease apart some trivial associations that arise from population biases or cellular heterogeneity. Specifically, the authors posit that by integrating two separate DNAm datasets, one can more readily identify biological sources of variation that are common across populations. Examples of such factors include age, sex, and cellular heterogeneity. Formally, CONFINED is a modified version of Canonical Correlation Analysis (CCA) for finding factors that jointly capture variability across two datasets. The authors show promising results in recovering age, sex, and cellular heterogeneity when the data is further impacted by batch effects.

Overall, the approach sounds interesting and results are promising. However, I think in the current form, the analysis is too superficial on several fronts:

1) the user is yet again left with the main problem of choosing at least 3 hyper-parameters. The current results do not paint a comprehensive picture about relevance and sensitivity of results to these parameters. A more thorough assessment is needed to guide the user, otherwise I don't foresee the method to be of wide usability.

*We thank the reviewer for the comments. Notably, all previous reference-free methods suffer from the same obstacle, generally there is a set of hyperparameters that has to be chosen based on prior knowledge or model selection approaches (for instance, in PEER one needs to choose the number of PEER factors as well as a number of inference parameters). It is often the case, that the exact choice of the parameters is not critical, i.e., the algorithms are robust to this choice. This is indeed the case for CONFINED, based on a sensitivity analysis that we now added to the manuscript based on the reviewer’s comment, as we describe below.*

*First, we show through a new sensitivity analysis in the revised manuscript that the method is generally robust to the choice of correlation threshold (Supplementary Methods; Figure S10, page 43). Across a large range of values (nearly across the interval of 0.5-1.0 out of 0.0-1.0), CONFINED was consistently accurate in its estimate of cell-type composition. We also note that this hyperparameter can be interpretable in terms of the “variability explained”, i.e., when set at different values one get different interpretation of the results.*

*Second, in the original manuscript we presented a sensitivity analysis to the number of features used. We have added a discussion about the choice of t in the Discussion (page 12) More generally, different choices of t will result in different types of sources of variation, i.e., more or less sparse, and therefore there is no ‘good’ or ‘bad’ choice of this parameter.*

*Lastly, the issue of choosing “k,” or the number of components to include in a model, is a general question for all reference-free methods and is not specific to CONFINED. For all methods, we note that increasing the number of components can only increase the R2 in our evaluations, however increasing the number of components also increases the risk of overfitting. We therefore suggest using a low number (e.g. 6 or 10) of CONFINED components as people often do in EWAS with principal components (Rahmani et al. Nat Methods 2016; Barfield et al. Genet Epidemiol 2014). We have added some guidance on usage to the Discussion section of the revised manuscript (page 13).*

2) Comparison with other methods suffers from the common problem of "lack of optimization" of those other methods (see below).

* Comparison to other methods. I found the comparison with existing method unsatisfactory in a typical way: CONFINED has at least 3 parameters that the authors play with, to ensure adequate performance, but it's not clear if the same level of attention was placed in adjusting the parameters of the other methods. For example, PEER has several parameters for balancing fit vs sparsity vs number of factors included.

*We thank the reviewer for bringing attention to this section. Though we did not make this explicit in the original text, we attempted to optimize each method’s performance. We have since added further information on our usage of the other methods to the revised Methods section of the text (page 16). Notably, many of the methods have the same number or more parameters than CONFINED. For example, PEER requires the number of factors, the number of iterations and a number of inference parameters; PMA also requires the number of factors, the number of iterations, and a number of other sparsity parameters.*

*In the case of PMA, we followed the authors’ code and used their cross-validation function to estimate optimal parameters, which, as the reviewer mentions balances the fit of the model by optimizing the sparsity. In the case of PEER we simply used the code in the authors’ example in their github wiki. We also followed the authors’ recommendations for optimizing the sparsity parameter and feature-selection steps of ReFACTor. In addition to the above, in the revised version of the manuscript, we also tried each of the methods using the top 1,000 to 10,000 most variable sites (with a step size of 1,000) for a more fair comparison (similarly to how was done in Houseman et al. BMC Bioinf. 2016) . The text in the page 16-17 in the Methods now reads: “When we induced sparsity in PMA, PEER and NNMF, the methods' performance were generally lower than when using no sparsity. In terms of R2 , we describe the results when using 10,000 sites and no sparsity respectively: R 2 = .47 as opposed to .54, R 2 = .49 PMA PEER compared to .52, R 2 = .49$ instead of .54. ReFACTor benefited most from sparsity and had NNMF 2 the highest performance when using 2,000 sites RReF =.79.”*

3) More data is needed to convince the user that CONFINED factors are relevant beyond the three obvious variables (age, sex, and cellular heterogeneity) that are typically easy to recover by existing methods in the absence of noise. I'll elaborate below.

*To show that our method is finding replicable sources of variability, we now include in the revised supplementary analyses of an additional blood-blood pair (Supplementary Methods pages 39,40), a brain-brain pair (Supplementary Methods page 26), and an adipose-adipose pair (Supplementary Methods page 27). Importantly, it is only possible to show the method’s performance for sources of variability that are known and in some form measurable. For the sources of variability that are unknown or unmeasurable, there is no ground-truth or means to evaluate our method against other methods. For these reasons, we have shown how CONFINED performs across multiple pairs of tissue types, as well as across situations in which datasets of different tissue types are paired together. In each case, CONFINED has captured the known biological variability (e.g. cell-type composition, sex, age, BMI). Further, the enrichment analyses (Results page 7; Supplementary Methods pages 29-31) and clustering experiment (Results page 11) provide evidence that CONFINED is in fact finding replicable biology.*

*Related to above, the authors seem to suggest that by using the same number of latent factors, the different approaches are put on similar footing for comparison (e.g., Figure 3). But this is clearly not true: the number of factors mean very different things, e.g., in sparse PCA, 10 PC would explain much fewer amount of variance, compared to 10 PCs in standard PCA. Likewise, whether or not 10 PEER factors have the same "explanatory power" as 10 CONFINED factors depend on sparsity level and other parameters in PEER.*

*While we agree with the reviewer that the factors generated by each method may have different meanings, most often these factors are introduced into a linear model, and the number of factors correspond to the number of degrees of freedom. We thus believe that this is indeed a fair comparison. The variance explained, suggested by the reviewer, is only one possible measure, and it is unclear how this is related to downstream analyses. If at all, sparse methods and CCA should capture less variance than PCA and PEER, and it may be argued that for this reason one should allow more factors to CONFINED.*

*It was not clear to me how the comparison in Figure 2 was done. Specifically, how were the parameters set and optimized?*

*We apologize and agree with the reviewer that this was not clear. We mention above and in the revised manuscript (Methods section page 16) how we optimized other methods and hoped to set up a more fair comparison. We attempted to give fair attention to each of the previous methods to try and optimize their performance.*

* Since CONFINED is using two datasets, whereas the other methods compared to use one dataset, it's not possible to distinguish whether the improved performance comes from the method itself or addition of new data. Thus the comparison to PCA applied to concatenated data is key for teasing these two apart. However, I found the comparison in this section superficial and lacking details. For instance, in CONFINED CCA is done twice (once to find t probes (feature selection) and once to construct a final set of latent variables from those selected probes). I suspect that the feature selection step is helping the approach, and if similar procedure was used for the "concatenated PCA", the results would be more comparable.

*We apologize that this was not clear in the original submission, but we should first emphasize that the comparison in this case was to sparse PCA (ReFACTor), which also performs a similar two-step algorithm, i.e. a feature-selection step (using PCA) followed by a second PCA on the selected features. In the original manuscript we did run ReFACTor’s full algorithm on the concatenated data, i.e., we used both datasets, which results in a fair comparison with CONFINED, as it uses the two datasets. Furthermore, we argue that CCA has an inherent advantage due to its multi-stage approach which would be analogous to saying that when running PCA, one first takes the product of a matrix with itself and then finds the eigenvectors of this matrix (so two steps). Clearly, one only has to care about the fact that the output of the algorithm is only given after the last step. Analogously, CONFINED has two steps, but the feature selection part is simply one of the steps of the algorithm.*

*We have made further clarifications to this issue in the revised manuscript (Supplementary Methods; Section S6 page 33).*

Minor:

* This statement needs clarification "In these experiments, we considered only known shared biological sources of variation and therefore excluded from our analyses sources of variation that may only appear in one dataset, e.g. patient status or brain location." Do you mean you explicitly modeled the effect of these variables, analogues to what is done in SVA to not over correct?

*We thank the reviewer for the suggestion and have attempted to make the sentence less ambiguous. We meant to say that since CONFINED only looks for biological variability that is present in both datasets, we did not evaluate its performance on sources of biological variability that were only known to be present in one of the datasets. The sentence now reads as follows: “As CONFINED looks for only shared biological sources of variation, we excluded from our evaluations sources of variation that may only appear in one of the datasets, e.g. patient status.”*

* I found the Gene Ontology section not that useful. It would be more informative to compare GO analysis between methods, otherwise its hard to say if the enrichment levels are interesting/different or typical in some way.

*We appreciate the reviewer’s concern and apologize that our intention was unclear. The enrichment analysis was not meant to find novel or atypical biology about the feature selection, but rather to suggest biological validity to the features that CONFINED selects. Importantly, there are unknown or unmeasurable sources of variability in methylation datasets. Since we can only evaluate our method on known or measurable sources of variability, we wished to provide further evidence for the strength of our method. To do so, we show through the enrichment analysis that by inducing sparsity, our method prioritizes CpG sites that are inherent to the shared b iology between two datasets. Thus, a comparison to other methods is irrelevant in this context.*

**Second round of review**

**Reviewer 1**

The new paragraph at lines 92 – 100: This paragraph is a clear statement of the potential of the method. However, could this paragraph finish with a simple sentence or two on what one could do with “captured” signal? It may be a little unclear what “capture” refers to and this word is extensively used. i.e. capture to use, or capture to throw away, or context dependent use of the components?

Line 238: please quantify this result. There does appear to be some diminution of the separation, even if it is not large.

A few words explaining the results seen in Supplement S7 – in that supplement section – would be helpful.

(In the response to reviews, I'm not sure that the reference to Figure S4 is accurate.)

**Reviewer 2**

The authors have addressed my questions/comments.

**Authors Response**

**Point-by-point responses to the reviewers’ comments:**

*We’d like to once more thank the reviewers for their insight and comments throughout this project. Your time and effort are very much appreciated, and we believe have made the manuscript of significantly better quality.*

Reviewer #1: The new paragraph at lines 92 – 100: This paragraph is a clear statement of the potential of the method. However, could this paragraph finish with a simple sentence or two on what one could do with “captured” signal? It may be a little unclear what “capture” refers to and this word is extensively used. i.e. capture to use, or capture to throw away, or context dependent use of the components?

*We have added brief commentary to the end of this paragraph, it reads as follows:*

*"In short, one could include the components generated by CONFINED to model their effects or interaction with the methylation in, for example, an EWAS, or instead remove the signal captured by the components prior to studying dataset-specific variability such as a treatment effect on a subset of individuals."*

Line 238: please quantify this result. There does appear to be some diminution of the separation, even if it is not large.

*We have added some commentary as well as a brief comparison between the two to the revised manuscript. The closing sentence now reads, “In the scenario where the treatment effect was a dominant source of variability, using CONFINED as a preprocessing step did not obstruct the ability to distinguish between those who received treatment and those who did not (the correlation between the treatment group and the first two PCs changed from .429 to .414)”*

A few words explaining the results seen in Supplement S7 – in that supplement section – would be helpful.

*We added to the revised manuscript the following sentences explaining the results: “Using the concatenated data as input to CONFINED performed much better than when using separate matrices as input into the sparse multiple CCA implementation. Further, the results using the concatenated datasets with CONFINED were consistent to those reported when using the datasets separately with CONFINED.”*
